# Time-space dynamics of income segregation in the city of Milan

**DOI:** 10.1093/pnasnexus/pgaf283

**Published:** 2025-09-05

**Authors:** Lavinia Rossi Mori, Vittorio Loreto, Riccardo Di Clemente

**Affiliations:** Centro Ricerche Enrico Fermi, Via Panisperna 89/A, Rome 00184, Italy; Physics Department, Università di Roma Tor Vergata, Via della Ricerca Scientifica, 1, Rome 00133, Italy; Centro Ricerche Enrico Fermi, Via Panisperna 89/A, Rome 00184, Italy; Sony Computer Science Laboratories Rome, Joint Initiative CREF-Sony, Centro Ricerche Enrico Fermi, Via Panisperna 89/A, Rome 00184, Italy; Physics Department, Sapienza University of Rome, Piazzale Aldo Moro 2, Rome 00185, Italy; Complexity Science Hub, Josefstädter Strasse 39, Vienna A 1080, Austria; Complex Connections Lab, Network Science Institute, Northeastern University London, London E1W 1LP, United Kingdom; ISI Foundation, Via Chisola 5, Turin 10126, Italy

**Keywords:** mobility, GPS, income segregation, social mixing, urban planning

## Abstract

Traditional approaches to urban income segregation focus on static residential patterns, often failing to capture the dynamic nature of social mixing at the neighborhood level. We leverage high-resolution location-based data from mobile phones to capture the interplay of three different income groups (high, medium, and low) based on their daily routines. The three income groups define a novel 3D space embedded in the temporal dynamics of urban activities, which we propose as a framework to analyze social mixing. This framework offers a more detailed perspective on social interactions, closely linked to the geographical features of each neighborhood. While nighttime residential patterns show high segregation, the working hours foster inclusion, with the city center showing heightened levels of interaction. As evening sets in, leisure areas emerge as potential facilitators for social interactions, depending on urban features such as public transport and various Points Of Interest. These characteristics significantly modulate the magnitude and type of social stratification involved in social mixing, underscoring the significance of urban design in bridging or widening socio-economic divides.

Significance Statement We investigate income segregation by analyzing the space-time dynamics of urban mobility using high-resolution mobile phone data. We introduce a new methodology to capture the time-varying social mixing of different income groups at the neighborhood level. The study examines how this social interaction changes throughout the day and is influenced by factors like public transportation accessibility, and the diversity and quality of local attractions. We applied this framework as case of study in the city of Milan revealing that neighborhoods with better transportation options and more diverse attractions promote greater social inclusion, particularly during evening activities.

## Introduction

Cities are vibrant organisms in constant evolution, continuously reshaped by economic, technological, and social forces (1). These dynamics shape the form and function of urban neighborhoods, influencing where individuals live, work, and interact (2). While technological advances and economic developments have redefined these environments, the benefits have not been uniformly distributed across cities (3) or neighborhoods (4). This inequitable allocation of opportunities (5) and services (6) leads to socio-economic disparity (7).

A recent study (8) has shown that residents in diverse residential contexts often experience limited exposure to varied social contacts due to the spatial and social confines of their neighborhoods. This highlights a disparity in opportunities for social interactions, despite the mobility afforded by urban living, particularly in areas of high residential segregation. Wealthier neighborhoods often enjoy better access to essential services, such as healthcare (9), education (10), and job opportunities (11), resulting in income-based urban segregation and limited interaction between different income groups (12). The growing income disparity (13) further compounds these inequalities, leading to spatial divisions within urban areas. These divisions go beyond mere physical boundaries (14), influencing how people interact with their environment, affecting mobility patterns (15), and social encounters (16).

The study of income segregation has expanded from an initial focus on residential patterns (17) to a more dynamic perspective that includes workplace and third places (18)—public spaces where people spend leisure or community time dynamics. This evolution reflects our changing understanding of segregation as a multidimensional phenomenon that manifests across various urban spaces and temporal scales (19). Research now extends beyond residential neighborhoods to include workplaces (20), educational institutions (21), and leisure spaces (22). The workplace, in particular, has emerged as a critical domain for understanding segregation dynamics, often functioning as a social bridge that fosters interaction among people from different urban areas (23).

This extension allows for a more comprehensive understanding of segregation dynamics, accounting for individuals’ diverse activities across various urban environments, studied at various spatial scales—from Points of Interest (POIs) (24), such as restaurants, grocery stores, museums, to streets (25) and neighborhoods (26). However, it is essential to recognize that while these elements are crucial, they offer only a microcosmic perspective on urban segregation dynamics. A street or a single point of interest represents just a fragment of a larger picture. The intricate interplay of more expansive neighborhoods, vital community hubs, and interconnected transport systems emerges as a central theme in understanding broader integration patterns and division within urban settings (27). Furthermore, by examining larger urban expanses, we often unearth deep-rooted socio-economic disparities and historical and cultural aspects, which frequently underpin the complexities of urban segregation.

The longitudinal aspect of the spatial dimension of urban dynamics is pivotal, but the temporal aspects offer equal insights. The same urban spaces may exhibit diverse social interactions at different times, raising the following question. If individuals are socially integrated in the spatial dimension, does this integration persist or transform across the temporal dimension? The dynamics of mobility within cities can foster social inclusion, but the extent of this inclusion varies with time and the distinct characteristics of the neighborhood (28). For example, some neighborhoods maintain a stable mix of income groups throughout the day, contributing to a consistent level of social inclusion. Conversely, others show variable degrees of social mixing depending on the time of the day: they might offer an inclusive atmosphere during the day, facilitating interactions among diverse social groups, but revert to a state of higher segregation after nightfall.

Everyday mobility normally serves as a mechanism for potential inter-group contacts, which when disrupted can reinforce existing patterns of segregation (29). However, research has also shown that even when physically mobile, residents of disadvantaged neighborhoods may remain socially isolated from more affluent areas (30).

The challenge is to dynamically observe these changes and understand the topological characteristics that can promote more inclusive neighborhoods. Using Location-Based Services (LBS) (31) trajectory data, we explore urban dynamics to capture the broader dimensions of income segregation. We analyze the relationship between income segregation and neighborhood topological features by examining the POIs that provide urban services within the same geographical space and the mixing of people in different time bands. To describe how citizens interact with the urban texture, we leverage trajectory LBS data from 94,000 users in Milan city over 10 months, integrated with a dataset from rent as a proxy for income (16). Through this comprehensive data integration, we gain insights into the hourly mobility patterns of residents.

Central to our analysis is the notion of “income triade,” which represents the distribution of the three income groups in the city. This triade allows us to capture the change of social mixing, indicating whether there is a balanced representation of all income groups (perfect mixing) or dominance by a single group (complete segregation).

We group and classify neighborhoods based on city structure-dependent features such as the efficiency of public transport, category diversity, or median price, defined by Accessibility, Livability, and Attractivity (ALA clustering). When we overlay the social mixing patterns—defined through the income triade—with the summarized urban fabric characteristics from ALA clustering, we capture the neighborhood level of income segregation.

We find that the magnitude of segregation is influenced by the neighborhood features represented in the ALA cluster, and the time of day. The variety of facilities a neighborhood offers and their utilization by different income groups at different time bands significantly impact the levels of segregation. We find a high level of spatial segregation at night due to residential segregation but observe increased social mixing during the day when residential segregation relaxes. Neighborhoods exhibiting interaction with middle-income groups appear to be more inclusive, as individuals from these groups commonly attend places frequented by low- and high-income groups.

Additionally, our study incorporates temporal dynamics into the segregation analysis, offering a novel framework to assess temporal mixing at the neighborhood level. This approach acknowledges that neighborhoods and their socioeconomic profiles do not remain static throughout the day but rather fluctuate depending on daily rhythms and human activities. By examining these temporal shifts, we distinguish three groups—inclusive, mixed, and segregated—and study the dis-similarity between these groups and those determined by the ALA metrics.

While established patterns of residential segregation and workplace integration provide important baseline, our analysis uncovers more complex dynamics that challenge conventional assumptions about urban centrality and infrastructure quality. By incorporating temporal dimensions and neighborhood characteristics, we reveal how high-quality urban spaces can exhibit “dual functionalities”—inclusive during work hours but exclusive during leisure periods—and how medium-quality neighborhoods can demonstrate unexpected social inclusivity despite infrastructure limitations.

Finally, through regression analysis, we could identify the key neighborhood features driving inclusivity and high social mixing. We find that the most influential characteristics of a neighborhood are its accessibility in terms of public transportation and diversity of amenities in category and price.

## Results

To comprehensively characterize the dimensions of segregation, we present a set of space-time metrics. The purpose is to capture the intricate spatial and temporal patterns that shape divisions within urban environments. The new metrics, rooted in a combination of topographical, socioeconomic, and human mobility data, provide a robust framework for understanding the subtle intricacies of urban interactions and disparities.

### Data

Leveraging individual Location-Based Service (LBS) trajectories, our study encompasses 650,000 users over 10 months, utilizing anonymized, high-resolution mobile location pings from the metropolitan area of Milan—with a population of 7,400,000 million (OECD 2006^a^). We detect the daily stay locations (Supplementary Note 1) using the time duration of 20 minutes and a spatial radius of 200 m as the Hariharan and Toyama algorithm (32). These locations are then classified into three categories: home, work, and third places. The identification of home and work locations is based on the analysis of each user’s periodic daily activities (33) (refer to Supplementary Note 1). We define third places as any stops that are neither home nor work. These are typically public spaces where individuals engage in leisure or community activities (18). Post-data cleaning (refer to Figs. S1 and S2), our sample size narrows to 368,625 with 103,329 users in Milan city, which represents approximately 10% of Milan city population.

To capture the users’ whereabouts linked to the social mixing by income stratification, we coupled the users’ residential area, extracted from the LBS, with rental data for Milan metropolitan area from Caasa.it^b^ for the period September 2022 to June 2023 (elaboration in Supplementary Note 2 and Fig. S5). As rent price near the home location has been proven a good proxy to infer users’ income (16), each user is associated with the average of the 10 closest home rent values within a 200-m radius of their home location extracted from LBS. Users inferred income is consistent with census data on median income levels^c^ with 0.89 of Pearson Coefficient at ZIP code level (see Fig. S6). In the second phase of data cleaning, we limited our analysis to the core zone of Milan, Milan city, where we identified 94,000 users with valid income assignments derived from rent data (for further details, please refer to Supplementary Note 2 and Fig. S7). We observed no spatial bias in the dataset with a robust correlation—Pearson coefficient = 0.88—between the users and the official census population in each section defined by ISTAT^d^ (see Fig. S8).

To effectively capture the behavioral differences in visited locations across various income groups and measure economic segregation levels within the city, we utilize k-means to classify individuals into three income groups: low, medium, and high (Fig. S9). Our decision for a tripartite division was influenced by methodological considerations and the objective to present a clear yet encompassing representation of socio-economic strata (see Supplementary Note 4 for details). To portray the interplay of social mixing dynamics within urban spaces, we leverage the three income clusters to create a 3D income vector space, the *income triade*  I. We represent each income group with a basis vector (see Supplementary Note 4, Equation S1). This framework allows us to classify users with a 3D vector representation.

Neighborhood structure has an impact on the social dynamic of the urban encounters, the points of interest locations (34), the street topology (35), and the urban space can affect our daily routines and social activities (36). A methodological challenge in studying these dynamics lies in defining what constitutes a “neighborhood”—a concept that often lacks precise spatial boundaries (37). Traditional approaches rely on various boundary definitions: administrative divisions (ZIP codes, census tracts), geographical features (rivers, major roads), socio-economic characteristics, or historical districts. However, these definitions typically suffer from inconsistent sizes, arbitrary delineations, and the modifiable areal unit problem (38), which can significantly influence segregation measures and obscure fine-grained interaction patterns.

To address these challenges, we employed a hexagonal tessellation approach (39), which provides a standardized spatial framework independent of preexisting administrative or geographical boundaries. This tessellation allows for consistent spatial sampling, merging the benefits of full coverage (akin to a square grid) with uniform spacing (see Methods section for details). After evaluating different resolutions, we selected a 300-m hexagon grid that optimally balances granular pattern detection with statistical robustness (see Supplementary Note 3, Figs. S10–S13). Throughout this study, the terms “hexagon” and “neighborhood” are used interchangeably to refer to these standardized spatial units.

**Fig. 1.**
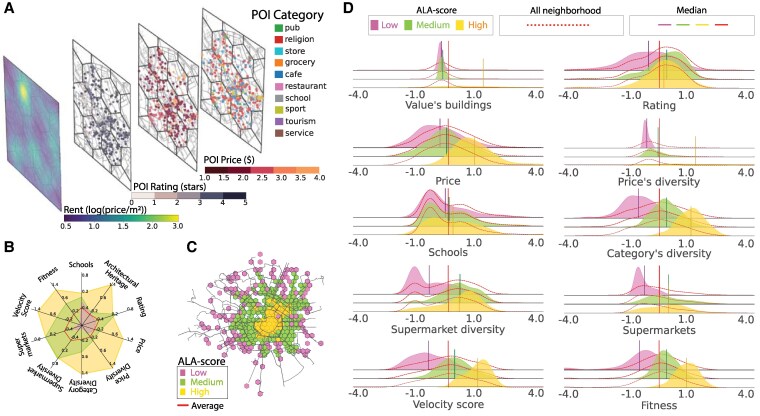
ALA clustering of neighborhoods in Milan city. A) Panels showcase the distribution of rent per square meter, reviews, price, and categories of POIs across selected central hexagons representing individual neighborhoods. B) Spatial map of Milan city illustrating cluster distribution, created using OpenStreetMap data: central regions predominantly exhibit high ALA metric values, whereas the periphery is characterized by medium and low ALA values. C) Comparative visualization of the median ALA metrics within each cluster against the average of all neighborhoods, depicted in red. D) Z-score distribution of various ALA metrics, including architectural heritage, rating, price, price diversity, schools, category diversity, supermarket diversity, supermarket count, velocity score, and fitness. Each cluster is denoted by a distinct color, with vertical lines representing its median value (see the legend). The overall neighborhood distribution, irrespective of clusters, is represented by the dotted red line, while the red vertical line signifies the median of all neighborhoods. Maps in panels (A) and (C) were created using Python with OpenStreetMap data, available under ODbL.

City topology and neighborhood characteristics influence our movement patterns and interactions with both individuals and the urban fabric (40). In this context, points of interest are pivotal to identifying services and opportunities available within these neighborhoods (41). We garner information across Milan from Google Places API^e^ (see Supplementary Note 5 and Fig. S27 for the category distribution) on ∼400,000 verified venues, including their latitude, longitude, amenity category, pricing, and reviews in Milan for the year 2022 (Fig. 1A).

### ALA-clustering

To capture the main feature that encourages social inclusivity within urban environments, we need to quantify neighborhood attributes integral to social mixing. While traditional methods lean on residents’ perceptions (42) or census data (43), our approach analyses neighborhoods based on their topographical and geographical traits, providing a more quantifiable and consistent means of characterization. These data can be easily captured, updated, and compared across different regions and cities (44).

Building on recent developments in urban metrics research, we introduce an integrated framework with three components: Accessibility, Livability, and Attractivity (ALA). This integration of previously separate measurement dimensions represents an advancement over studies that have examined individual aspects in isolation.

Accessibility defines the ease of reaching a location with public transportation (45) (details in Supplementary Note 6). To quantify the Livability metric, we consider both the number and diversity of schools and supermarkets and the architectural value of a building (see Supplementary Note 6). This metric primarily serves to understand how essential services, such as schools or supermarkets, are distributed across different income groups, thereby highlighting accessibility disparities within the urban landscape. How a neighborhood is diverse in terms of POIs by type, price, and reviews plays a key role in determining a neighborhood’s attractivity (46). We describe Attractivity in terms of a neighborhood’s Fitness (47) (see Supplementary Note 6)—capturing both amenity diversity and uniqueness—as well as price diversity, median pricing, and reviews. The fitness calculation employs a bi-adjacency matrix between neighborhoods and POI categories (Fig. S30), where neighborhoods with high fitness scores contain diverse or unique combinations of amenities that enhance their attractiveness to visitors from different income groups. The low correlation between these metrics (Fig. S15) confirms they capture distinct aspects of urban structure, providing a comprehensive characterization of neighborhood qualities.

We detect three distinct clusters using the k-means algorithm to the z-score normalization of the ALA metrics (Fig. 1B). The rationale behind choosing three clusters for this analysis is detailed in the Supplementary Note 4, with elbow method analysis (Fig. S23) confirming the optimal number of clusters. Alternative configurations with 4 and 5 clusters (Figs. S24 and S25) are evaluated, and cluster similarity analysis (Fig. S26) validates the robustness of our three-cluster solution. Each cluster reflects a different level of neighborhood quality (Fig. 1C). The cluster composed of more central hexagons, labelled “high,” exhibited the highest values for almost all metrics, translating to greater accessibility, a wider variety of POIs, and a higher uniqueness of categories. In contrast, the peripheral cluster recorded the lowest values, indicating limited choices of POIs, schools, and restaurants and reduced accessibility from the rest of the city (Fig. 1D). Schools appear uniformly accessible across all neighborhoods, a direct outcome of national legislative mandates that ensure equitable access to education (48). This uniformity starkly contrasts the differentiation observed in other attributes, underscoring the influence of policy-driven urban planning.

The observed radial pattern in Fig. 1B confirms residential income segregation witnessed in many European cities (49), underscores the uneven distribution of amenities and opportunities among citizens, which favors the city center to the peripheries. Spatial distributions of singular ALA metric are in Figs. S28 and S29.

### Social mixing

While the Gini index (50) has served as a valuable tool in segregation studies, it compresses the complexity of social interactions into a single number. This compression is useful for broad comparisons; however, it masks the specific composition of groups contributing to segregation. Consequently, distributions with different underlying income patterns might receive similar Gini values, which can lead to misleading interpretations when tracking how segregation changes over time.

To evaluate how the characteristics of neighborhoods relate to income-based social mixing over time, we define a time-sensitive metric in the income triade, the Neighborhood Income Activity, ${\vec{A}}_{h,t}$, at a specific time *t* in a given neighborhood *h*. This vector aggregates the activities of all users from different income groups in (h,t) and enables us to track the changes in social mixing across the city (see Supplementary Note 7, Equations S8 and S9). To ensure a fair comparison across neighborhoods, an L1 normalization transforms the vector components into proportions.

The three coordinates of the Neighborhood Income Activity are in the Income Triade space $e_{H}$, $e_{M}$, and $e_{L}$, so ${\vec{A}}_{h,t}=(0,0,1)$ represents the exclusive presence of users belonging to the “Low” income group in the neighborhood *h* at time *t*. Conversely, ${\vec{A}}_{h,t}=(0.33,0.33,0.33)$ represents an inclusive neighborhood that is visited equally by each income group.

**Fig. 2.**
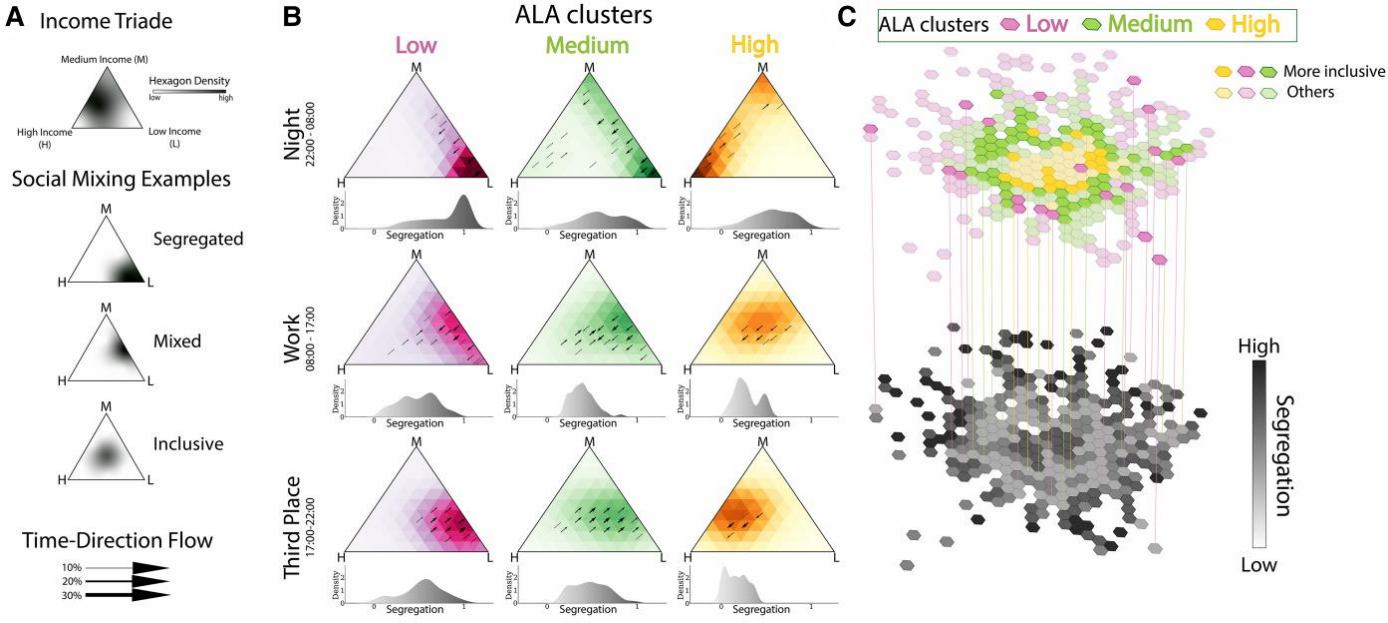
Income triade analysis: segregation dynamics. A) Legend and interpretation of the income triade. The triangle’s vertices represent the three income groups: high, medium, and low. The stronger the color, the higher the density of neighborhoods in that configuration. A neighborhood’s proximity to a vertex indicates a predominant visitation from that specific income group. When a neighborhood’s visitation is evenly distributed among the income groups, it positions near the triangle’s center. From top to bottom, the examples illustrate a segregated neighborhood predominantly visited by the low-income group (situated at the bottom vertex), a mixed neighborhood frequented by both low and medium incomes (located on the right edge), and an inclusive neighborhood with equal representation from all three income groups (centered within the triangle). To analyze segregation temporally, we employ arrows. The direction of these arrows forecasts the neighborhood’s segregation trend in the subsequent time slot, while their thickness represents the density of neighborhoods experiencing a similar pattern. B) Each column demonstrates the spatial shift in segregation across the ALA clusters: high, medium, and low. In contrast, each row presents a temporal dimension, representing different time bands: home during night-time (22:00–08:00, working hours (08:00–17:00), and leisure activities in the evening (17:00–22:00). Below each triangle, we report the histograms of the segregation values, as measured in terms of the Gini coefficient (see Supplementary Note 9), for all neighborhoods belonging to the same ALA cluster and the same time band. C) A two-tiered map representation created with Python libraries: the upper layer emphasizes the most inclusive neighborhoods with brighter colors, while the other hexagons are opaque. On the lower tier, the color gradient reflects the level of segregation calculated using the Gini index. All hexagons from the top tier transition into a light gray on the bottom layer, indicating low segregation.

Unlike single-value segregation metrics, this visualization immediately reveals the specific income composition of each neighborhood. We can immediately distinguish between neighborhoods showing medium–high income mixing versus those with medium–low income mixing—distinctions that would be lost in a single segregation score. The framework also captures temporal dynamics through directional arrows, making social composition changes immediately perceptible. In Fig. 2A, we provide examples of aggregate behaviors of the neighborhoods. When the income triade is denser at the bottom right (see Fig. 2A, second triangle), a set of neighborhoods at that time will be mostly visited by the low-income group. In the second scenario, where the density is greater on the right edge, we see a collection of mixed neighborhoods frequented by both low and medium incomes (see Fig. 2A, third triangle). Finally, the fourth triangle of Fig. 2A is denser at the center, indicating that all three income groups equally visit the neighborhoods.

Figure 2B draws the heat map of the activity of each neighborhood within the income triade, normalized based on user density, across different ALA clusters and temporal frames. This visualization underscores how neighborhoods in varying ALA clusters become the focal points of activities for citizens with distinct income stratification at different times of the day.

Our temporal analysis follows a structured progression to reveal how segregation patterns transform through three fundamental contexts of urban life: during the night (22:00–08:00), where we analyze only the stops made at home; throughout the day (08:00–17:00), focusing solely on stops at workplaces; and in the evening (17:00–22:00), where our attention is on third places.

The three sequential temporal windows are designed to capture distinct aspects of urban social dynamics. We can trace how segregation patterns transform throughout the day: from the structural constraints of residential segregation at night, through the institutional mixing that occurs during work hours, to the voluntary social interactions that emerge in evening leisure settings.

By analyzing only home stops at night, we gain insight into the first layer of our temporal analysis, the patterns of static residential segregation, establishing the baseline socio-economic geography when mobility is minimal. Moving through the day’s progression, we shift our focus to workplace environments. Our approach aligns with Ellis (20), who observed less segregation in workplace areas compared to residential neighborhoods, suggesting more diverse interactions during work hours. Similarly, Candipan (51) highlighted the significant variation in urban racial segregation between residential and employment settings, reinforcing the importance of considering both domains in studies of urban segregation to explore the role of employment settings as a connection for social interaction among diverse income groups.

In the evening, our focus on third places shows how people from different economic backgrounds mingle in social settings outside their homes and workplaces. This approach underscores the urban social dynamics, revealing how the nature and location of interactions shift distinctly across different times of the day.

Within each representation of income distribution, the arrows pinpoint the gradient shifts in the subsequent time section, providing a predictive insight into temporal social dynamics. Spatial segregation peaks at night, largely attributed to the inherent residential income disparities (17), exacerbating the differences in the home location of different income groups.

The pink cluster, predominantly inhabited by the low-income group, contrasts with the green cluster—home to both low and medium incomes—and the yellow one that hosts the high and medium-income groups (see Fig. S16). However, a more diverse interaction appears during the day: residential segregation is relaxed across all clusters, albeit with varying intensities. In the first row of Fig. 2B, the arrows indicate a transition from stringent segregation to an increased social income mixing. During work hours, people from different income groups converge in their workplaces, highlighting the role of work in temporarily bridging economic gaps (23). The yellow cluster benefits most from the inclusive effects of work hours. One potential interpretation is that individuals with lower incomes might be seeking employment opportunities within high ALA cluster areas due to the prospect of better social capital and “economic connectedness” (52). While these areas can offer substantial benefits, the trade-off frequently materializes in increased commuting times for these individuals and consequent worse quality of life (53).

The evening transition, characterized by third-place activities such as leisure amenities, exhibits a drift in the income triade towards the periphery, outlining a more pairwise social mixing with interactions between adjacent income groups. This trend leans towards residential segregation in the night hours.

The income triade permits us to capture the subtler interplay of income-based social mixing during transition hours. Specific attributes render a neighborhood attractive not just to its inhabitants but to outsiders as well. This allure is contingent on the amenities a neighborhood offers, leading to distinct behaviors in the three ALA clusters, and it resonates differently across income groups. The high ALA cluster emerges as more inclusive, although the presence of medium income is more conspicuous than that of the low income—they exhibit limited mobility from their primary neighborhood. Interestingly, the data show that the middle-income group visits areas that are common to both low- and high-income groups, as evidenced by the dense distribution in the middle-income corner across all three clusters (Fig. 2B and Figs. S19 and S20).

We can employ the Gini function (50) to have a quantifiable metric to gauge the income social mixing in the neighborhood. The Gini coefficient (Supplementary Note 8, Equation S10) ranges between 0 (implying perfect mixing) and 1 (indicating complete segregation), serving as a quantifiable reflection of our observations in the ternary plot.

The distributions positioned below each triangle in Fig. 2B emphasize the contrasting behaviors of the three ALA clusters. While the low ALA cluster invariably exhibits pronounced segregation in all temporal situations, the other two clusters display greater inclusivity. Having analyzed the behavior of the three income groups using fixed time windows, our focus now shifts to the temporal evolution of the neighborhood through the Gini index. This dynamic lens offers a more granular insight into income-based social interactions, and observing how segregation ebbs and flows throughout the day enhances our comprehension of day-long shifts in income segregation.

We direct our focus toward analyzing the dynamics of third places, extending our analysis across the entire week, examining data over 48-h periods, weekdays and weekends. This choice is motivated by two key considerations: first, defining work activities during weekends presents methodological challenges as most citizens are not engaged in traditional workplace activities; second, our preliminary analysis showed that home and work locations exhibit relatively stable segregation patterns with limited temporal variation (Figs. S21 and S22), while third places demonstrate the most dynamic social mixing behaviors. This analysis is illustrated in the two-level map (Fig. 2C), where we observe the distribution of segregation within the city (below) and identify the most inclusive hexagons (above) with the presence of all clusters. This heterogeneity suggests a more complex underlying division of hexagons, different from the one done with the ALA metrics.

### Social mixing temporal evolution

**Fig. 3.**
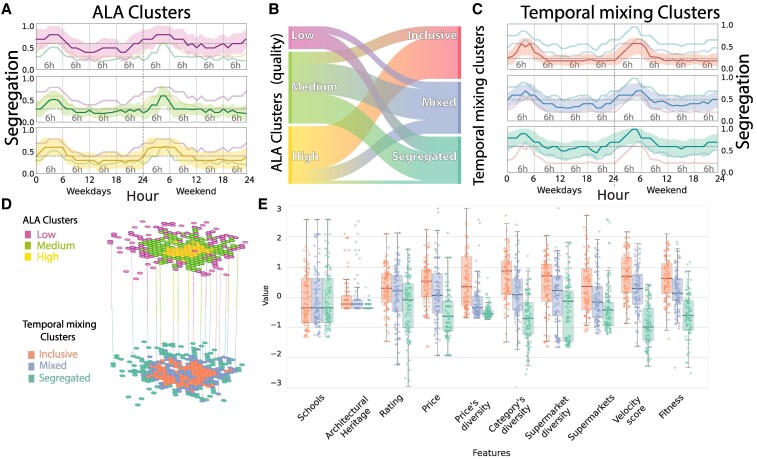
Interplay of ALA Features in Determining Income Segregation. A) Segregation trends within the ALA clusters over weekdays and weekends. The solid line denotes the median, and the variability is captured within the shaded region representing the SD. The median lines from the other two clusters are also shown for comparison in each cluster. B) Visual representation of the neighborhood distribution across the ALA and temporal mixing clusters. C) Segregation trends for the newly defined clusters—inclusive, mixed, and segregated—are termed “temporal mixing clusters,” across weekdays and weekends. Again, the solid line represents the median, with the shaded region indicating the SD. The median lines from the other two clusters are also shown for comparison in each cluster. D) A two-tiered map representation: the upper layer highlights the ALA clusters’ spatial distribution, and the segregation profiles are lower. E) Box plot and jitter plot illustrate the distribution of the zscore for the 10 ALA metrics across the three temporal mixing clusters.

We now characterize each neighborhood by a unique time series of Gini coefficients spanning 48 h: 24 on weekdays and 24 on weekends derived exclusively from third-place activities to capture the voluntary social interactions that drive temporal mixing patterns. Considering the topological classification of the city neighborhoods, we aggregate the daily evolution of the Gini coefficient through the lens of ALA clusters, observing a consistent pattern (see Fig. 3A). Neighborhoods with a low value in the ALA metrics show a stronger inclination towards segregation than those classified as medium and high.

This initial approach focuses solely on the city’s topology via the ALA clusters, thereby overlooking the potential similarity in the daily evolution of the Gini coefficients. To address this, we set aside the predefined ALA cluster classifications. Instead, we sought to create new clusters based solely on the temporal trends of the Gini coefficient for each neighborhood. Through this different approach, using k-means, we discern three distinct patterns: inclusive (red-top line), mixed (blue-center line), and segregated (green-bottom line) (as showcased in Fig. 3C). The decision to use three clusters is elaborated upon in the Supplementary Note 4.

The inclusive neighborhoods consistently register a lower Gini index throughout the day, while the segregated cluster deviates significantly from the other two.

The spatial distribution of the temporal clusters breaks from the radial patterns of the ALA clusters, implying a multifaceted urban interplay (as showcased in Fig. 3D). Remarkably, not all neighborhoods characterized by the same ALA metrics merge into the same temporal mixing cluster (Fig. 3B).

Our framework identifies shifts in the temporal mixing of citizens. In particular, we observe that high ALA neighborhoods in the city center exhibit two distinct temporal patterns: one maintains inclusivity throughout the day, while the other shows a ’dual functionality,’ being inclusive during working hours but becoming mixed or segregated at other times. High ALA areas that remain inclusive outside of work tend to have greater price and category diversity but lower architectural heritage values compared to those that shift toward temporal segregation (Fig. S31).

The inclusive neighborhoods form a ring-like pattern around the city center, suggesting that specific characteristics function as crucial “transition zones” that bridge socioeconomic divides (54). The features that most significantly distinguish these hexagons from central areas are more affordable prices and greater price diversity.

To provide a comprehensive understanding of the urban landscape and its influence on the temporal dynamics of social mixing, we studied the distribution of the ALA metrics in relation to the temporal clusterization (Fig. 3E). Each temporal mixing cluster manifests a unique fingerprint when mapped onto the urban topology. Interestingly, there is a wide distribution difference in “price diversity,” “velocity score,” and “fitness” by temporal clusters. These metrics play a pivotal role in shaping its temporal inclusivity and are strictly related to the fabric of a neighborhood—such as type of amenities, accessibility, and availability of services.

We utilized a regression model to identify the key factors linked to social mixing and inclusivity to evaluate how different aspects of “accessibility,” “livability,” “attractivity,” and population density influence urban segregation, specifically analyzing the median value of segregation across the 48-h period.

Observing spatial autocorrelation in our data, we incorporated a Spatial Lag model. For a detailed exposition of the regression analyses, including the application of ordinary least squares (OLS) (Table S1) and error models (Table S2). These extended analyses offer comprehensive insights into the statistical relationships governing urban segregation in Milan city.

Adopting a Lindeman, Merenda, and Gold (LMG) (55) approach, we discerned that “accessibility” and “attractivity” heavily sway the segregation trends, accounting for ∼55% of its variance, while ’livability’ and population density play a lesser role (Table 1). A deeper probe using LASSO revealed pivotal features: Velocity Score, Fitness, Median Price, and Price Diversity. Subsequent application of an OLS model confirmed the statistically significant negative impact of these features on segregation (details in Table 1). Conversely, architectural landmarks, also of statistical significance, contribute to increased segregation.

**Table 1.** Regression results of the spatial lag model for predicting segregation using combinations of ALA metrics.

Group	Variable	Only Accessibility	Only Livability	Only Attractivity	Only Density	All Together	LASSO
Accessibility	Velocity Score	− 0.76***				− 0.42***	− 0.42***
Livability	Schools		− 0.08			− 0.02	
	Supermarkets		0.01			0.05	
	Supermarket diversity		− 0.32**			− 0.02	
	Architectural heritage		0.31**			0.19***	0.21***
Attractivity	Fitness			− 0.38**		− 0.22**	− 0.30***
	Category’s diversity			− 0.35*		− 0.15	
	Price’s diversity			− 0.20*		− 0.14*	− 0.18*
	Price			− 0.50***		− 0.21***	− 0.23***
	Rating			− 0.23		− 0.07	
Population	Density				− 0.03*	− 0.03	
Autocorrelation	Segregation	0.24***	0.33***	0.27***	0.26***	0.24***	
$R^{2}$		0.56	0.31	0.57	0.34	0.65	0.62

Significance levels: *P < 0.05, **P < 0.01, ***P < 0.001.

This result highlights the city’s inequality, revealing that not all citizens can enjoy its architectural marvels, and in fact, in areas where there are more buildings of significant architectural value, segregation is higher. These results underscore the importance of considering multiple factors when attempting to understand and address urban segregation. Providing accessible, diverse, and attractive neighborhoods can foster social interactions and reduce segregation within cities.

## Discussion

In recent years, urban researchers and policymakers have turned their attention to the pressing issue of income segregation in cities (56), recognizing its implications for access to essential services and opportunities. Income segregation is not just about the surroundings of citizens’ residences but critically about *how much* individuals from varied economic backgrounds interact.

Our method overcomes the conventional 2D representation of segregation via the Gini coefficient. By introducing the income triade, we transition into a 3D space that offers a more detailed perspective on social mixing in Milan city. This innovative approach does not just indicate the extent of segregation but also its composition, revealing the proportion of each income group present during specific time frames, from nightly spans to working hours and leisure periods. The income triade helps uncover how different income levels affect access to services and reveals hidden interaction patterns between socioeconomic groups often missed in traditional analyses.

Our findings improve understanding of urban segregation by exposing dynamics throughout the day. Rather than simply confirming residential segregation and workplace integration, we identify “dual functionalities” in high-quality central areas and persistent “transition zones” in medium-quality neighborhoods, despite their infrastructure limitations. These temporal patterns highlight that urban inclusivity relates not only to overall location or quality of services but also to specific neighborhood features.

Key factors influencing social mixing include public transportation accessibility, which fosters interaction between people from different socioeconomic backgrounds, and amenity diversity, which provides opportunities for all income groups (25). In contrast, high-value architecture drives up property prices and living costs, leading to economically homogeneous communities that exclude lower-income groups and reinforce urban segregation.

We acknowledge the limitations of using hexagonal grid cells as proxies for neighborhoods. Traditional neighborhoods evolve through complex historical, cultural, and social processes that create meaningful boundaries not captured by geometric tessellation (57, 58). However, hexagonal tessellation ensures consistency allowing us to detect fine-grained socio-spatial dynamics that might otherwise remain obscured when using administrative boundaries of varying sizes.

While our study encompasses a comprehensive set of metrics to analyze urban segregation, we acknowledge that certain factors, such as environmental pollution, were not included. Although not considered in our current analysis, the impact of pollution on urban livability and segregation presents an intriguing avenue for future research. Integrating environmental data could significantly enrich our understanding of how urban dynamics are influenced by ecological factors, adding depth to the study of urban segregation.

Regarding the geographical scope of our study, we recognize the limitations inherent in focusing solely on Milan city due to data availability. This boundary issue is a common challenge in urban research, and our study’s confinement to Milan city highlights the need for broader data access and analysis in future studies. Expanding research to encompass areas beyond city limits could provide a more complete picture of urban dynamics and segregation patterns, addressing a critical gap in the field of urban studies. The methodology developed, however, holds potential for broader application and can be adapted to other urban settings. Although the core methodology is expected to remain consistent, different urban environments may reveal unique segregation patterns based on local conditions.

Beyond its current application, our methodology is versatile. It can be recalibrated to investigate other forms of segregation, such as those based on ethnicity or other sociodemographic parameters, offering a more holistic view of urban inclusivity. Although our research paints a detailed portrait of Milan city urban dynamics over 10 months, further research could extend this framework to longer timeframes, potentially revealing gentrification patterns.

Our research aligns with the growing academic interest in urban segregation. It not only offers an in-depth analysis of Milan city urban dynamics but also presents a valuable framework for both researchers and policymakers. Our tools are poised to offer profound urban insights, guiding both academic discussions to further analyze the intricate urban interactions and policymaking towards more inclusive urban futures.

## Methods

Our analytical framework integrates spatial and temporal dimensions to provide a comprehensive characterization of urban socioeconomic segregation patterns.

### Income

Our approach leverages a comprehensive rental dataset that includes property types ranging from single rooms to villas (Fig. S3), ensuring representation across the full spectrum of Milan’s housing market (Fig. S4). We classified users into three income groups (high, medium, low) using k-means clustering of residential rental prices following established methodological approaches in socioeconomic stratification (59). While conventional approaches in income segregation studies often employ fixed thresholds such as the Eurostat standard (60% below median for low-income, 140% above for high-income) (29), these rigid boundaries proved unsuitable for Milan city unique income distribution. Fixed thresholds resulted in severely imbalanced group sizes, with very small low-income groups or high income groups that would limit statistical power for segregation analysis. K-means clustering offers three key methodological advantages: (i) adaptive sensitivity to the natural distribution of income within the specific urban context, (ii) statistically optimized group sizes that facilitate robust segregation analysis, and (iii) data-driven boundaries that better reflect Milan city socioeconomic fabric. We validated our approach through elbow method analysis (Fig. S17) and silhouette score analysis (Fig. S18), confirming k-means produced the most cohesive and well-separated income groups. This tripartite division enables the creation of our novel income triade visualization framework, which maps social mixing patterns within a 3D vector space. In this representation, the vertices of a triangle correspond to the three income groups, and the position within the triangle indicates the proportional mix of these groups in each neighborhood (Fig. S16).

### Spatial dimension

For our spatial analysis, we implemented a hexagonal tessellation with 300-m sides. We test multiple grid resolutions (200–700 m) to determine optimal size, evaluating each resolution’s capacity to capture meaningful socio-spatial patterns while maintaining sufficient population per unit for statistical validity (see Supplementary Note 3, Figs. S10–S13 for detailed comparison). To ascertain the robustness of our findings, we conducted validation tests comparing segregation measures across different grid resolutions, finding high correlations (Pearson coefficients >0.90) between the 300-m grid and alternative resolutions, as illustrated in Fig. S14. This confirms that our results are not artifacts of the specific grid resolution but reflect consistent underlying urban patterns.

### Temporal dimension

To capture the temporal dimension, we developed a multilayer temporal analysis framework that follows a progression through different activity patterns across the day.

Our approach examines three distinct scenarios: nighttime residential patterns (22:00–08:00), daytime workplace dynamics (08:00–17:00), and evening leisure activities (17:00–22:00). Each layer builds upon the previous: The nighttime analysis establishes baseline distribution of socioeconomic groups across neighborhoods. Workplace analysis then shows how daily mobility transforms these patterns, with employment serving as a mechanism for social mixing. Finally, evening analysis of third places provides insight into voluntary interaction patterns—where people actively choose to spend leisure time. This sequential approach distinguishes between static residential segregation and potentially more inclusive dynamics during working hours and leisure time, capturing a complete 48-h cycle of urban social mixing patterns.

This approach allows us not only to confirm expected patterns of residential segregation at night and increased mixing during work hours, but crucially enables identifying neighborhoods that deviate from expected patterns. By examining the full 48-h temporal profile of segregation metrics, we can distinguish between superficially similar quality neighborhoods that nonetheless demonstrate fundamentally different temporal inclusivity patterns.

## Supplementary Material

pgaf283_Supplementary_Data

## Data Availability

The mobile phone data that support the findings of this study were acquired by Sony from Cuebiq. Restrictions apply to the availability of these data, which were used under license for this study. However, researchers interested in accessing similar datasets can contact Cuebiq to discuss data acquisition for academic research through multiple pathways: **Social Impact Program:** Researchers can explore Cuebiq’s Social Impact program at https://cuebiq.com/social-impact/. **Commercial Academic Access:** Researchers can request a demonstration and discuss purchasing data for academic research by filling out the form at https://cuebiq.com/demo/ or by contacting Cuebiq directly at socialimpact@cuebiq.com. **Data for Good Program:** Information about Cuebiq’s broader data sharing initiatives can be found at Cuebiq - Data for Good program. To facilitate replication of our key findings, we have made available aggregated data and analysis code in our GitHub repository. These materials include processed datasets that preserve privacy while enabling reproduction of our main results, along with detailed documentation of our analytical procedures. Additional datasets used in this study are publicly available: venue location and category data were obtained via Google Places API, and rental price data were collected from publicly accessible listings on www.caasa.it using automated data collection methods detailed in the Supplementary materials. The website to explore our results can be accessed via Riccardo Di Clemente website.

## Notes

a
https://www.oecd-ilibrary.org/urban-rural-and-regional-development/oecd-territorial-reviews-milan-italy-2006_9789264028920-en (Accessed: 2025 September 1).

b
https://www.caasa.it/ (Accessed: 2025 September 1).

c
https://www1.finanze.gov.it/finanze/analisi_stat/public/index.php? (Accessed: 2025 September 1).

d
https://www.istat.it/it/archivio/104317 (Accessed: 2025 September 1).

e
https://developers.google.com/maps/documentation/places/web-service/overview?hl=en (Accessed: 2025 September 1).

## Preprints

This manuscript was posted on a preprint: https://arxiv.org/abs/2309.17294.
