# Supplementary material for: Time-space dynamics of income segregation in the city of Milan
Source: PNAS Nexus. 2025 Sep 5;4(9):pgaf283. doi: 10.1093/pnasnexus/pgaf283 (PMC12449691; doi:10.1093/pnasnexus/pgaf283)
Supplement: pgaf283_Supplementary_Data [file pgaf283_supplementary_data.pdf]

Supplementary Materials for *Time-space dynamics of income segregation in the city of Milan*

Lavinia Rossi Mori[1,2], Vittorio Loreto[3,4,1,5], and Riccardo Di Clemente[6,7*]

[1] *Centro Ricerche Enrico Fermi, Via Panisperna 89/A, 00184, Rome, Italy*
[2] *Physics Department, Università di Roma Tor Vergata, 00133, Rome, Italy*
[3] *Sony Computer Science Laboratories Rome, Joint Initiative CREF-Sony, Centro Ricerche Enrico Fermi, Via Panisperna 89/A, 00184, Rome, Italy*
[4] *Physics Department, Sapienza University of Rome, Piazzale Aldo Moro 2, 00185, Rome, Italy*
[5] *Complexity Science Hub, Josefstädter Strasse 39, A 1080 Vienna, Austria*
[6] *Complex Connections Lab, Network Science Institute, Northeastern University London, London, E1W 1LP, United Kingdom.*
[7] *ISI Foundation, 10126, Torino, Italy*
[*] *corresponding author: riccardo.diclemente@nulondon.ac.uk*

28 August 2025

Supplementary Notes

1 Supplementary Note 1 – Location Data **3**
1.1 Extracting stays . . . 3
1.2 Identifying important places . . . 3
1.2.1 Identifying home and work . . . 3
1.3 Data Sanitisation . . . 4

2 Supplementary Note 2 – Income and Rent Dataset **4**
2.1 Rent dataset . . . 4
2.2 Income Representativeness . . . 5
2.3 Population Representativeness . . . 5

3 Supplementary Note 3 – Hexagonal Grid **5**

4 Supplementary Note 4 – Clusterisation **5**
4.1 Assigning Economic Status . . . 5
4.2 Income Group Clusterisation . . . 5
4.3 City Clusterisation . . . 6

5 Supplementary Note 5 – Venues **6**

6 Supplementary Note 6 – ALA Metrics: Assessing Urban Accessibility, Livability, and Attractivity **7**
6.1 Accessibility . . . 7
6.1.1 Velocity Score . . . 7
6.2 Liveability . . . 8
6.3 Attrattivity . . . 8

7 Supplementary Note 7 – Neighbourhood Income Activity and Normalisation 10

8 Supplementary Note 8 – Gini Coefficent 10

9 Supplementary Note 9 – Regression 10

Supplementary Figures

Figure S1 User Distribution in Milan city and Surrounding Areas . . . 17
Figure S2 Segregation Profile With Different Filter . . . 17
Figure S3 Categories of Rental Properties . . . 18
Figure S4 Rent Price Distribution . . . 18
Figure S5 Rent Data as a Proxy of Income . . . 19
Figure S6 Rappresentativness of Income . . . 19
Figure S7 Data Representativeness in top 30 Municipalities . . . 20
Figure S8 Representativeness of Data in Each ISTAT Section. . . . 20
Figure S9 Kmeans Income Groups . . . 21
Figure S10 POIs Distribution Maps . . . 22
Figure S11 Boxplot in Each Grid . . . 23
Figure S12 Distribution in Each Grid . . . 24
Figure S13 Segregation Profile for Each Grid . . . 25
Figure S14 Correlation for the Hexagonal Grids . . . 25
Figure S15 Correlation Between the ALA Metrics. . . . 26
Figure S16 Ternary Difference . . . 27
Figure S17 Elbow Method Analysis . . . 27
Figure S18 Silhouette Score Analysis . . . 28
Figure S19 Square-form Ternary Plot for 4 Income Groups . . . 28
Figure S20 Pentagon-form Ternary Plot for 5 Income Groups . . . 29
Figure S21 Home Segregation Profile . . . 30
Figure S22 Work Segregation Profile . . . 31
Figure S23 Elbow Method Analysis . . . 31
Figure S24 Analysis Results for 4 Clusters . . . 32
Figure S25 Analysis Results for 5 Clusters . . . 33
Figure S26 Cluster Similarity . . . 33
Figure S27 Categories Distribution . . . 34
Figure S28 Map Distributions for all the Hexagons . . . 34
Figure S29 Map Distributions . . . 35
Figure S30 The (bi-)adjacency matrix . . . 35
Figure S31 High-ALA neighborhoods . . . 36

Supplementary Equations

Eq. S1 Income Triade Basis . . . 5

Supplementary Tables

Table S1 OSL Model Regression . . . 12
Table S2 Spatial Error Model Regression . . . 13

1 Supplementary Note 1 – Location Data

The LBS (Location-Based Services) data utilised in this study have been shared by Sony Computer Science Laboratories - Rome, under a non-disclosure agreement (NDA). This type of data has been widely used and validated in numerous studies [1, 2, 3]. The data were collected from 650,000 anonymous mobile phone users over a span of 10 months from March 2017 to December 2017, who have opted-in to provide access to their location data anonymously through a GDPR-compliant framework. In adherence to privacy compliance, all the stops belonging to the Education, Health, and Religion categories were removed from the dataset. The data were processed and analysed in a secure and privacy-compliant environment to ensure adherence to the terms of the NDA and relevant data protection laws.

From the GPS trajectory in the LBS data, we extract 24 million stops (*pings*), each with latitude and longitude with an accuracy of 20m and the associated timestamp.

1.1 Extracting stays

The open-source Python software toolkit [4] extracts location in three steps:

- Stop detection: all the locations where users spent at least 20 minutes within a distance of 200 meters from a given point.
- The stops can be aggregated in location, which means that all the stop clusters refer to the same point. Adopting the method defined in [5], the DBSCAN algorithm is used to group together points given their local spatial density, with two parameters: the maximum linked distance ϵ, and s, the minimum number of neighbours within distance ϵ for a point to be regarded as a core point.
- At the end of the analysis, all stops belonging to the same location: each stop is assigned to the medoid of the sequence.

1.2 Identifying important places

Every site has a significance, namely home, and work.

1.2.1 Identifying home and work

Algorithm 1.1: 3.2.1: home and work detection (user features)

```
Input: Stops = (UID, lat, long, time)
Output: Assigned home and work for each user
Function home detection:
    for users in Users do
        stops between 9 p.m. and 6 a.m.;
        stops close together;
        cluster centroids and assign home;
        fraction of home visits n_home;
        if 0.2 < n_home < 0.8 then
            take user;
        else
            drop user;
Function work detection:
    for users in Users do
        stops between 8 a.m. and 5 p.m., no weekends;
        most visited stops are work;
```

To derive meaningful insights from each location, it is imperative to associate them with significant places relevant to users, especially their homes and workplaces. We utilise the algorithm presented in 1.1 for this purpose.

For our geographical scope, we adopt the Organisation for Economic Co-operation and Development (OECD) definition of urban territories [6]. The OECD introduces two distinct boundaries: a core region, in this case the metropolitan city of Milan, and an expansive vicinity named the Functional Urban Area (FUA), with the commuting zone surrounding the city. We take all the stops in both areas.

1.3 Data Sanitisation

A rigorous data sanitisation process was employed to guarantee statistical reliability. To isolate and exclude tourists and sporadically connected users [7, 8], we selected only those who had at least 15 unique active days with at least one recorded position. To further refine our dataset and ensure a confident understanding of users' home locations, we focused on users who return home at least one day to four. This filtering resulted in a reduction of 60% of our initial user dataset [9, 2] . This sanitisation leads us to consider 368,625 individuals in the Milan region, with 28% (103,329) of users in Milan city (Fig S1).

Subsequently, we refined our user sample by implementing filters that allowed us to select individuals based on their average daily ping frequency, specifically targeting those with an average of 1 to 3 pings. This step ensured a more focused and consistent analysis. In performing these comparative analyses, we found no substantial variances in the resulting data, confirming the robustness of our findings across different user engagement levels (as illustrated in Figure S2, which we will discuss in further detail). This process not only reinforces the reliability of our data but also highlights the adaptability of our methodology to different user activity levels.

By assigning work, from 8 a.m. to 5 p.m. without weekends, a significant portion of restaurant workers are excluded. To address this, the work classification was assigned to all stops made very frequently and for more than two and a half hours at a restaurant.

2 Supplementary Note 2 – Income and Rent Dataset

Income data in Italy is typically available at the broader ZIP code level, which necessitates a more granular approach for detailed analysis. In Milan, the area covered by ZIP codes varies significantly, ranging from about 1.5 km^2 for central districts to as large as 18 km^2 for outer areas. To achieve a finer detail in approximating individual economic statuses, we employed house rent prices per square meter as a proxy [9]. This method allows for a more precise estimation of economic conditions at a smaller spatial scale, aligning our analysis with the urban dynamics of Milan. Data from the website Caasa [10], derived from various Italian real estate platforms, provided one year of rent prices per square meter within Milan's OECD-defined region, i.e. both the core, the municipality of Milan, and the municipalities that are part of Milan's commuting zone, the FUA. [6].

2.1 Rent dataset

We obtained data on various types of properties: Figure S3 shows the different categories of rental properties, and we focused on habitable residences with the following distribution of prices, as depicted in Figure S4.

The majority of municipalities in the FUA region would leave all users without a valid assignment, and even the 30 municipalities with the largest populations would have many NaNs (see Figure S7). Figure S5 also indicates that areas outside Milan exhibit sparse data and lower Pearson correlation compared to inner-city regions. Consequently, we concentrated our analysis within Milan city, the core, where rent serves as a reliable income indicator, resulting in a dataset of 94,000 users.

2.2 Income Representativeness

To evaluate the representativeness of our dataset in accurately reflecting the income distributions across different ZIP codes in the city, we conducted a correlation analysis. Specifically, we compared the median income values assigned to each user in our dataset with the median actual income reported for each ZIP code. This comparison aims to validate the accuracy of our income assignment methodology and is visually depicted in Figure S6.

2.3 Population Representativeness

To assess the goodness of our dataset to capture the real population distributions across the city representatively, we measured the correlation between the number of users and the number of citizens in the ISTAT sections (Figure S8).

3 Supplementary Note 3 – Hexagonal Grid

To achieve a macroscopic representation of the city, we employed a hexagonal tessellation strategy, as described in [11]. The choice of grid size influenced the number and diversity of Points of Interest (POIs) encompassed within it. Larger grids, with sides measuring 600 and 700 meters, yielded a less representative portrayal of the city, as depicted in (Figure S10). The results show that analysing cities at broader scales (e.g., districts or ZIP codes) can miss the change in human interactions and their diverse urban settings.

We cluster with the ALA metrics the remaining grid dimensions. The data presented in figures S11 and S12 indicate that a grid with 500-meter sides distinguishes clusters most distinctly. Nonetheless, the evaluation of segregation profiles, as presented in Figure S13, showed a flat pattern for larger grids. Balancing between classifier quality and the segregation profile trend, we opted for a grid with sides measuring 300 meters. We check the robustness of the results at the zip code level. In Figure S14, each point represents the median of segregation of the grids present in a zip code, which is a highly aggregated measure but with a significant correlation.

4 Supplementary Note 4 – Clusterisation

4.1 Assigning Economic Status

Neither dividing the income into equal quantiles nor splitting the users into equal parts would accurately represent the diversity in income distribution. Dividing income into equal quantiles resulted in disproportionate representation, with values showing an uneven distribution of users across low, medium, and high-income groups (e.g., low: 92991, medium: 1451, high: 66). Kmeans instead resulting in three distinct income clusters depicted in Figure S9.

We leverage the three income clusters to create a 3D income vector space, the *income triade* $\mathbb{I}$. We represent each income group with a basis vector denoted as:

$$\mathbf{e}_H = \begin{bmatrix}1\\0\\0\end{bmatrix}, \quad \mathbf{e}_M = \begin{bmatrix}0\\1\\0\end{bmatrix}, \quad \mathbf{e}_L = \begin{bmatrix}0\\0\\1\end{bmatrix} \qquad \text{(Eq. S1)}$$

where $\mathbf{e}_H$, $\mathbf{e}_M$, and $\mathbf{e}_L$ represent the High, Medium, and Low-income groups, respectively.

4.2 Income Group Clusterisation

We examined different cluster sizes to determine the most appropriate number of income groups for our analysis. Based on the elbow method, as illustrated in the Figure S17, we considered the partitioning into three clusters. This decision was further validated through silhouette score analysis (Figure S18), which assessed the consistency within clusters versus the separation between them.

Despite relying on these methodological approaches, we also aimed to ensure that our results were consistent with the actual movements of individuals. To this end, we analysed movement patterns with both four (Figure S19) and five (Figure S20) income groups, observing coherent results in both cases. However, dividing the dataset into three income groups—low, medium, and high income proved to be most effective. This segmentation not only captured distinct patterns of economic segregation but also maintained clarity and simplicity in the model, facilitating our investigation into social mixing dynamics.

To portray the interplay of social mixing dynamics within urban spaces, we leverage the three income clusters to create a 3D income vector space, the *income triade* $\mathbb{I}$. We represent each income group with a basis vector denoted as:

$$\mathbf{e}_H = \begin{bmatrix} 1 \\ 0 \\ 0 \end{bmatrix}, \quad \mathbf{e}_M = \begin{bmatrix} 0 \\ 1 \\ 0 \end{bmatrix}, \quad \mathbf{e}_L = \begin{bmatrix} 0 \\ 0 \\ 1 \end{bmatrix} \tag{Eq. S2}$$

where $\mathbf{e}_H$, $\mathbf{e}_M$, and $\mathbf{e}_L$ represent the High, Medium, and Low-income groups, respectively.

Users vector

Every user k, with $k = 1, ..., K$, where K is the total number of users, can be mapped into the *Income Triade*. The user vector, $\vec{u_k}$, is described as:

$$\vec{u_k} = \delta(u_k, \vec{u}) \tag{Eq. S3}$$

The function $\delta(u_k, \vec{u})$ maps the user k to a three-dimensional vector, where the component corresponding to the income category of the user k is 1, and all other components are 0.

4.3 City Clusterisation

For city-wide clusterisation, the elbow method, as depicted in Figure S23, indicated three as the optimal number of clusters. To gain a deeper understanding of Milan's spatial dynamics, we explored configurations with higher cluster counts, specifically 4 and 5 clusters, as presented in Figures S24 and S25, respectively. While these configurations yielded coherent results, we observed an overlap in the characteristics of the clusters, suggesting that the increase in cluster count did not necessarily lead to a more distinct separation of urban features.

The decision to adopt k=3 was driven by our objective to maintain clarity in our analysis and effectively capture the diverse urban landscape of Milan. The three-cluster model provided a balanced representation, offering meaningful differentiation without overcomplicating the spatial representation.

We employed different clustering methods to ensure the robustness of our findings. Specifically, for the ALA metrics we used k-means and agglomerative clustering [12] techniques for clusterization. After clusterization we assessed the similarity of the resulting clusters obtaining the Fowlkes-Mallows score [13] of 0.73. For the Temporal Mixing we used k-means, agglomerative, and spectral [14] clustering techniques for clusterization. After clusterization,

we assessed the similarity of the resulting clusters obtaining the Fowlkes-Mallows score of 0.84.

5 Supplementary Note 5 – Venues

POI Datasets

We use the Google API to collect data on the Points of Interest (POIs). We start by selecting a point k=(lat, long) on the maps. We fetch the first 50 POIs nearby, generating a correspondence hexagonal area of interaction from the point A_k. We collect the attributes - "latitude", "longitude", "category", "price", and "rating" within the A_k for all the POIs, and we iterate the process to patch

all the city areas. We collected more than 400,000 POIs. Utilising the Google Place classifications, we categorised the venues frequented by individuals. For generic classifications, such as "point of interest", we referred to Bing for specific categorisation. If unavailable, these generic classifications were discarded. The venues were then grouped systematically according to our Taxonomy as illustrated in (Figure S27).

6 Supplementary Note 6 – ALA Metrics: Assessing Urban Accessibility, Livability, and Attractivity

Neighbourhood structure has an impact on the social dynamic of the urban encounters; the points of interest locations [15], the street topology [16], and the urban space [17, 18], can affect our daily routines and social activities [19]. These components are important in defining the neighbourhoods [20] as the geographically basic component that interlinks community and human interactions with urban texture [21].

To capture the main feature that encourages social inclusivity within urban environments, we need to quantify neighbourhood attributes integral to social mixing. While traditional methods lean on residents' perceptions [22] or census data [23], our approach analyses neighbourhoods based on their topographical and geographical traits, providing a more quantifiable and consistent means of characterisation. These data can be easily captured, updated, and compared across different regions and cities [24]. We introduce three novel metrics to systematically evaluate these neighbourhood characteristics: Accessibility, Liveability, and Attractivity (ALA).

Maps distributions for the ten ALA metrics are shown in (Figure S28, S29).

6.1 Accessibility

In the context of urban planning and development, accessibility is a key indicator of the degree of connectivity and inclusivity within a city's fabric [25]. In this case, it measures the ease with which individuals can reach a particular location from other parts of the city, reflecting the efficiency of the urban infrastructure in facilitating movement and interaction. A highly accessible city minimizes travel time and costs, thereby enhancing the quality of life for its inhabitants and contributing to a more vibrant and dynamic urban environment [26]. This aspect of urban design is crucial for ensuring that all city dwellers have equitable access to resources, services, and opportunities.

To quantify accessibility within urban settings, we leverage the velocity score [27], a measure of how quickly and efficiently one can access other parts of the city from a specific location. It is an indicator of urban reachability, reflecting the effectiveness of the transportation network in enabling residents to explore and interact with the city.

6.1.1 Velocity Score

The velocity score evaluates the efficacy of a city's transportation systems, strongly relying on the concept of isochronic maps. An isochronic map, as introduced by Galton [28], consists of isochrones centred at a given location λ. Each isochrone $I(\tau, (\lambda, t_0))$ demarcates areas that can be reached from λ within a travel time τ, starting at an absolute time t_0. The collective isochrones for varying τ values represent the isochronic map for λ at time t_0.

To understand the transportation spread from a starting point, we analyse the rate of isochronic expansion over time. Specifically, following [27], we consider the area $A(\tau, (\lambda, t_0))$ covered by the isochrone for a given τ. This area includes hexagons whose centres are attainable within time τ, and its growth is characterised by discrete jumps corresponding to hexagon centre acquisitions.

The calculation integrates all available public transportation modes (metro, bus, tram, urban trains) along with walking paths. We utilize GTFS (General Transit Feed Specification) [1] data provided by public transport companies, containing complete schedules and routes. We process the

[1] https://gtfs.org/

entire public transport network, including all stops and interconnections between different modes. The Intransitive Connection Scan Algorithm [27], also considers the possibility of walking up to 15 minutes to reach stops that are not directly connected.

Considering a circle with an area equivalent to the isochrone, its radius $\bar{r}$ represents the mean distance travelled in a random direction from the starting point:

$$\bar{r}(\tau, (\lambda, t_0)) = \sqrt{\frac{A(\tau, (\lambda, t_0))}{\pi}}$$

Consequently, the circular velocity $\bar{v}$ is:

$$\bar{v}(\tau, (\lambda, t_0)) = \frac{\bar{r}(\tau, (\lambda, t_0))}{\tau}$$

representing the expansion speed of a circular isochrone identical in area to the actual one.

The Velocity Score (v), as introduced in [27], is the average over times τ and t_0:

$$v(\lambda) = \frac{\sum_{t_0=6\text{am}}^{10\text{pm}} \int_0^\infty \bar{v}(\tau, (\lambda, t_0)) f(\tau) d\tau}{\sum_{t_0=6\text{am}}^{10\text{pm}} f(\tau) d\tau} \quad \text{(Eq. S4)}$$

where t_0 spans from 6 a.m. to 10 p.m. in 2-hour intervals, thus covering the entire public transport service day. The average over τ uses the empirical distribution $f(\tau)$ as a weight. It is worth noting, as pointed out in [27] that the choice of distribution for average travel times holds a degree of arbitrariness.

This methodology allows us to accurately capture the effectiveness of public transportation, considering not only the speed of individual modes but also waiting times, transfers, and pedestrian accessibility across the entire urban transport network.

6.2 Liveability

Neighbourhood liveability is influenced by many factors [29], three essential indicators of a neighbourhood's liveability and potential deprivation or prosperity are its educational accessibility [30], shopping options [31, 32], and architectural heritage [33]. To quantify the Liveability metric, we consider both the number and diversity of schools and supermarkets and the value of a building. We chose not to incorporate green spaces as an additional criterion for liveability, given their inherently dualistic nature [34]: they may provide benefits when well-maintained [35], but also evoke concerns and apprehension when in a degraded state [36]. Due to the absence of information regarding the condition of parks, we have opted to leave them out of our analysis.

6.3 Attrattivity

How a neighbourhood is diverse in terms of POIs by type, price, and reviews plays a key role in determining a neighbourhood's attractivity [37] and promoting social interaction [38]. We describe Attractivity in terms of a neighbourhood's Fitness [39] – capturing both amenity diversity and uniqueness – as well as price diversity, median pricing, and reviews. Such diversity can promote walkability [40], which not only has a positive impact on neighbourhood housing valuation but also on reducing neighbourhood crime and foreclosure rates [41].

Fitness and Complexity

Economic Complexity Framework The Fitness and Complexity framework, developed by Tacchella *et al.* [39], was originally designed to assess economic complexity based on the interaction between countries and products. This framework uses the Revealed Comparative Advantage (RCA) [42] as a foundational measure:

$$RCA^{(y)}_{cp} = \frac{M^{(y)}_{cp}}{\sum_{p'} M^{(y)}_{cp'}} \bigg/ \frac{\sum_{c'} M^{(y)}_{c'p}}{\sum_{c'p'} M^{(y)}_{c'p'}} \qquad \text{(Eq. S5)}$$

where M_{cp} represents the bi-adjacency matrix of the bipartite network between countries (c) and products (p), with elements equal to 1 if country c exports product p and 0 otherwise.

The Fitness F_c for country c and the complexity Q_p for product p are calculated through an iterative process:

$$\begin{cases} \tilde{F}^{(n)}_c = \sum_p M_{cp} Q^{(n-1)}_p \\ \tilde{Q}^{(n)}_p = \frac{1}{\sum_c M_{cp} \frac{1}{F^{(n-1)}_c}} \end{cases} \implies \begin{cases} F^n_c = \frac{\tilde{F}^{(n)}_c}{\langle \tilde{F}^{(n)}_c \rangle_c} \\ Q^n_p = \frac{\tilde{Q}^{(n)}_p}{\langle \tilde{Q}^{(n)}_p \rangle_p} \end{cases} \qquad \text{(Eq. S6)}$$

where $\langle \cdot \rangle$ indicates the average over the respective set, and initial conditions are $F_0 = Q_0 = 1$ for all countries and products. The framework employs a highly non-linear relationship: while developed countries export almost all products (providing limited information about product complexity), when an underdeveloped country exports a product, this strongly suggests the product requires low sophistication. Therefore, for a product to achieve high complexity, it must be produced only by highly competitive countries.

Framework Adaptation to Urban Context Building on this economic framework, we adapt the Fitness and Complexity approach to urban analysis. In [43], Juhász *et al.* demonstrated how economic complexity [44] can quantify the ability of urban locations to attract diverse visitors from various socio-economic backgrounds. Similarly, [22] found a noteworthy association between economic complexity and income inequality in urban contexts.

Urban Application: Neighborhoods and POI Categories In our urban adaptation, we replace the country-product relationship with a neighborhood-POI category relationship. We consider that individuals may choose to visit particular locations due to the availability of multiple amenities or unique features that are scarce in other areas, such as stadiums or cultural venues.

The analogy between neighborhoods-POI categories and countries-products becomes evident: many neighborhoods contain common points of interest that are less 'complex' (widely available), while unique and exceptional POIs are found in only a few areas. Therefore, a neighborhood with high fitness will be more attractive to individuals seeking diverse or unique features.

For our urban application, M_{hp} becomes the bi-adjacency matrix of the undirected bipartite network between hexagonal neighborhoods (h) and POI categories (p): its elements are 1 if neighborhood h contains at least one POI of category p, and 0 otherwise.

The fitness calculation follows the same iterative process as Equation Eq. S6, but now applied to neighborhoods and POI categories:

$$\begin{cases} \tilde{F}^{(n)}_h = \sum_p M_{hp} Q^{(n-1)}_p \\ \tilde{Q}^{(n)}_p = \frac{1}{\sum_h M_{hp} \frac{1}{F^{(n-1)}_h}} \end{cases} \implies \begin{cases} F^n_h = \frac{\tilde{F}^{(n)}_h}{\langle \tilde{F}^{(n)}_h \rangle_h} \\ Q^n_p = \frac{\tilde{Q}^{(n)}_p}{\langle \tilde{Q}^{(n)}_p \rangle_p} \end{cases} \qquad \text{(Eq. S7)}$$

Figure S30 illustrates the resulting bi-adjacency matrix between hexagonal neighborhoods and POI categories, sorted according to their fitness-complexity values. Neighborhoods with high fitness scores contain diverse or unique POI combinations that make them particularly attractive.

While Fitness and Category Diversity show high correlation (Figure S15), we retain both metrics in our analysis to capture the distinct concepts of uniqueness (fitness) and diversity (category count), which are both vital for understanding neighborhood attractivity.

7 Supplementary Note 7 – Neighbourhood Income Activity and Normalisation

For the initial distribution of incomes in the general population, we normalise each component of $\vec{A}_{h,t}$ using the total number of users in each income category. Denote these totals as N_H, N_M, and N_L for the High, Medium, and Low categories, respectively:

$$\vec{\tilde{A}}_{h,t} = \left(\frac{\sum_{k \in U_{h,t}} \delta(u_k, \vec{u})_H}{N_H}, \frac{\sum_{k \in U_{h,t}} \delta(u_k, \vec{u})_M}{N_M}, \frac{\sum_{k \in U_{h,t}} \delta(u_k, \vec{u})_L}{N_L} \right) \quad \text{(Eq. S8)}$$

Here, $U_{h,t}$ represents the set of users located in h at a time t, $\delta(u_k, \vec{u})_i$ represents the i-th component of the user vector, indicating whether user k belongs to income category i.

We then apply the L1 normalisation to obtain a proportionate distribution:

$$\vec{A}_{h,t} = \frac{\vec{\tilde{A}}_{h,t}}{\sum_i \vec{\tilde{A}}_{h,t,i}} \quad \text{(Eq. S9)}$$

8 Supplementary Note 8 – Gini Coefficent

We utilised the following metric, which is a function of the Gini coefficient[2] , to summarise the percentage of visiting a neighbourhood from the three different income groups throughout the hours of the day. 0 implies perfect mixing, and 1 indicates complete segregation.

$$G = \frac{\sum_{i=1}^{n-1} \sum_{j=i+1}^{n} |x_i - x_j|}{n}, \quad \text{(Eq. S10)}$$

where the terms x_i and x_j denote the proportion of users in these groups for a specific neighbourhood and the denominator serves as a normalisation factor. In the specific case of $n = 3$, i.e., the three income categories corresponding to the high, medium, and low-income groups, one has:

$$G = \frac{|x_1 - x_2| + |x_1 - x_3| + |x_2 - x_3|}{3} \quad \text{(Eq. S11)}$$

9 Supplementary Note 9 – Regression

High-quality neighborhoods (High-ALA) display strong temporal variance in social mixing - they become more inclusive during working hours but revert to higher segregation during evenings and weekends. This creates a 'dual personality' where these areas function as inclusive work centers during the day but transform into more exclusive spaces during leisure hours. Neighborhoods that maintain their inclusive status throughout all time periods have different feature profiles from those that degrade from inclusive to mixed status, see figure S31. The inclusive-maintaining areas show higher price diversity and category diversity but lower architectural heritage values.

Adopting a Lindeman, Merenda, and Gold (LMG) [45], in our analysis, we opted for the Spatial Lag Model over the Ordinary Least Squares (OLS) and the Spatial Error Model due to its capacity to explicitly incorporate spatial dependency in the regression equation. Our preference for the Spatial Lag Model over the Spatial Error Model was influenced by a combination of factors. Firstly, the Spatial Lag Model offered a more robust explanation of the data, as evidenced by a higher pseudo R-squared value. This indicated that the Spatial Lag Model accounted for a larger portion of the variance in the dependent variable compared to the Spatial Error Model.

Secondly, the analysis of residuals played a crucial role. The Spatial Lag Model effectively addressed the issue of spatial autocorrelation in the residuals. In spatial analysis, it is essential

[2] https://www.census.gov/topics/income-poverty/income-inequality/about/metrics/gini-index.html

not only to fit the model well to the observed data but also to ensure that the residuals (the differences between observed and predicted values) do not exhibit spatial autocorrelation. If spatial autocorrelation exists in the residuals, it suggests that some important spatial processes might have been omitted from the model. The Spatial Lag Model, in our case, showed better performance in this aspect, ensuring that the residuals were more randomly distributed without patterns of spatial autocorrelation, thus providing a more reliable and accurate representation of the spatial relationships in the data.

Group	Variable	Only Accessibility	Only Liveability	Only Attractivity	Only Population	All Together	LASSO
Accessibility	Velocity Score	-1.16***				-0.6***	-0.65***
	Schools		-0.08			-0.02	
Liveability	Supermarkets		0.01			0.01	
	Supermarket diversity		-0.32			-0.08	
	Architectural Heritage		0.31**			0.36***	0.37***
	Fitness			-0.38**		-0.19*	-0.29**
	Category's diversity			-0.35*		-0.11	
Attractivity	Price's diversity			-0.20*		-0.14*	-0.18*
	Price			-0.50***		-0.41***	-0.38***
	Rating			-0.23		-0.17	
Population	Density				-0.152***	-0.0295	
R^2		0.428	0.116	0.412	0.513	0.165	0.595

Table S1 Regression results of the OSL model for predicting segregation using combinations of ALA metrics.

Group	Variable	Only Accessibility	Only Liveability	Only Attractivity	Only Density	All Together	LASSO
Accessibility	Velocity Score	-0.75***				-0.59***	-0.58***
	Lambda	0.67***				0.63***	0.63***
	Schools		-0.08			-0.03	
Liveability	Supermarkets		0.14			0.14	
	Supermarket diversity		-0.09			-0.06	
	Architectural Heritage		0.10			0.11	0.10
	Lambda		0.88***			0.63***	0.63***
	Fitness			-0.18*		-0.07	-0.12*
	Category's diversity			-0.05		-0.03	
Attractivity	Price's diversity			-0.16*		-0.15*	-0.15*
	Price			-0.13*		-0.11*	-0.11*
	Rating			-0.01		-0.01	
	Lambda			0.76***		0.63***	0.63***
Population	Density				-0.01	-0.01	
	Lambda				0.65***	0.63***	0.63***
R^2		0.49	0.05	0.48	0.53	0.57	0.57

Table S2 Regression results of the Spatial Error model for predicting segregation using combinations of ALA metrics.

References

[1] Gergő Tóth, Johannes Wachs, Riccardo Di Clemente, Ákos Jakobi, Bence Ságvári, János Kertész, and Balázs Lengyel. Inequality is rising where social network segregation interacts with urban topology. *Nature Communications*, 12, 2021.

[2] Esteban Moro, Dan Calacci, Xiaowen Dong, and Alex Pentland. Mobility patterns are associated with experienced income segregation in large us cities. *Nature Communications*, 12, 2021.

[3] Zhuangyuan Fan, Tianyu Su, Maoran Sun, Ariel Noyman, Fan Zhang, Alex 'Sandy' Pentland, and Esteban Moro. Diversity beyond density: experienced social mixing of urban streets. *PNAS Nexus*, 2023.

[4] Enrico Ubaldi, Takahiro Yabe, Nicholas K. W. Jones, Maham Faisal Khan, Satish V. Ukkusuri, and Riccardo Di Clemente Emanuele Strano. Mobilkit: A python toolkit for urban resilience and disaster risk management analytics using high frequency human mobility data. *CoRR*, abs/2107.14297, 2021.

[5] Ramaswamy Hariharan and Kentaro Toyama. Project lachesis: Parsing and modeling location histories. *Lecture Notes in Computer Science (including subseries Lecture Notes in Artificial Intelligence and Lecture Notes in Bioinformatics)*, 3234, 2004.

[6] Delineating functional areas in all territories. `https://data.oecd.org/`.

[7] Santi Phithakkitnukoon, Zbigniew Smoreda, and Patrick Olivier. Socio-geography of human mobility: A study using longitudinal mobile phone data. *PLoS ONE*, 7, 2012.

[8] Lauren Alexander, Shan Jiang, Mikel Murga, and Marta C. Gonzalez. Origin-destination trips by purpose and time of day inferred from mobile phone data. *Transportation Research Part C: Emerging Technologies*, 58, 2015.

[9] Yang Xu, Alexander Belyi, Paolo Santi, and Carlo Ratti. Quantifying segregation in an integrated urban physical-social space. *Journal of the Royal Society Interface*, 16, 2019.

[10] Caasa. `https://www.caasa.it/m`.

[11] E. Ben-Joseph and D. Gordon. Hexagonal planning in theory and practice. *Journal of Urban Design*, 5, 2000.

[12] Fionn Murtagh and Pedro Contreras. Algorithms for hierarchical clustering: an overview, ii, 2017.

[13] E. B. Fowlkes and C. L. Mallows. A method for comparing two hierarchical clusterings. *Journal of the American Statistical Association*, 78, 1983.

[14] Andrew Y. Ng, Michael I. Jordan, and Yair Weiss. On spectral clustering: Analysis and an algorithm. 2002.

[15] Yang Yue, Yan Zhuang, Anthony G.O. Yeh, Jin Yun Xie, Cheng Lin Ma, and Qing Quan Li. Measurements of poi-based mixed use and their relationships with neighbourhood vibrancy. *International Journal of Geographical Information Science*, 31, 2017.

[16] Paul Dimaggio and Filiz Garip. Network effects and social inequality, 2012.

[17] Daniel Sauter and Marco Huettenmoser. Liveable streets and social inclusion. *Urban Design International*, 13, 2008.

[18] Qian Li, Caihui Cui, Feng Liu, Qirui Wu, Yadi Run, and Zhigang Han. Multidimensional urban vitality on streets: Spatial patterns and influence factor identification using multisource urban data. *ISPRS International Journal of Geo-Information*, 11, 2022.

[19] Vikas Mehta. Streets and social life in cities: a taxonomy of sociability. *Urban Design International*, 24, 2019.

[20] Taylor Shelton and Ate Poorthuis. The nature of neighborhoods: Using big data to rethink the geographies of atlanta‚Äôs neighborhood planning unit system. *Annals of the American Association of Geographers*, 109, 2019.

[21] David J. Madden. Neighborhood as spatial project: Making the urban order on the downtown brooklyn waterfront. *International Journal of Urban and Regional Research*, 38, 2014.

[22] Philip Salesses, Katja Schechtner, and César A. Hidalgo. The collaborative image of the city: Mapping the inequality of urban perception. *PLoS ONE*, 8, 2013.

[23] Jaap Nieuwenhuis, Tiit Tammaru, Maarten van Ham, Lina Hedman, and David Manley. Does segregation reduce socio-spatial mobility? evidence from four european countries with different inequality and segregation contexts. *Urban Studies*, 57, 2020.

[24] Cesar A. Hidalgo, Elisa Castaner, and Andres Sevtsuk. The amenity mix of urban neighborhoods. *Habitat International*, 106, 2020.

[25] Karst T. Geurs and Bert van Wee. Accessibility evaluation of land-use and transport strategies: Review and research directions. *Journal of Transport Geography*, 12, 2004.

[26] Martin Wachs and T. Gordon Kumagai. Physical accessibility as a social indicator. *Socio-Economic Planning Sciences*, 7, 1973.

[27] Indaco Biazzo, Bernardo Monechi, and Vittorio Loreto. General scores for accessibility and inequality measures in urban areas. *Royal Society Open Science*, 6, 2019.

[28] Francis Galton. On the construction of isochronic passage-charts. *Proceedings of the Royal Geographical Society and Monthly Record of Geography*, 3, 1881.

[29] Kostas Mouratidis. Neighborhood characteristics, neighborhood satisfaction, and well-being: The links with neighborhood deprivation. *Land Use Policy*, 99, 2020.

[30] Michael R. Greenberg. Improving neighborhood quality: A hierarchy of needs. *Housing Policy Debate*, 10, 1999.

[31] Belkis Cerrato Caceres and Jacqueline Geoghegan. Effects of new grocery store development on inner-city neighborhood residential prices, 2017.

[32] Alison A. Gustafson, Sarah Lewis, Corey Wilson, and Stephanie Jilcott-Pitts. Validation of food store environment secondary data source and the role of neighborhood deprivation in appalachia, kentucky, 2012.

[33] Waldemar Cudny and Hakan Appelblad. Monuments and their functions in urban public space. *Norsk Geografisk Tidsskrift*, 73, 2019.

[34] Perver K. Baran, William R. Smith, Robin C. Moore, Myron F. Floyd, Jason N. Bocarro, Nilda G. Cosco, and Thomas M. Danninger. Park use among youth and adults: Examination of individual, social, and urban form factors. *Environment and Behavior*, 46, 2014.

[35] Hanneke Kruizse, Nina van der Vliet, Brigit Staatsen, Ruth Bell, Aline Chiabai, Gabriel Muinos, Sahran Higgins, Sonia Quiroga, Pablo Martinez-Juarez, Monica Aberg Yngwe, Fotis Tsichlas, Pania Karnaki, Maria Luisa Lima, Silvestre Garcia de Jalon, Matluba Khan, George Morris, and Ingrid Stegeman. Urban green space: creating a triple win for environmental sustainability, health, and health equity through behavior change, 2019.

[36] Bonnie K.L. Mak and C. Y. Jim. Examining fear-evoking factors in urban parks in hong kong. *Landscape and Urban Planning*, 171, 2018.

[37] Sanaz Saeidi and Derya Oktay. Diversity for better quality of community life: Evaluations in famagusta neighbourhoods. *Procedia - Social and Behavioral Sciences*, 35, 2012.

[38] Arthur T. Row and Jane Jacobs. The death and life of great american cities. *The Yale Law Journal*, 71, 1962.

[39] Andrea Tacchella, Matthieu Cristelli, Guido Caldarelli, Andrea Gabrielli, and Luciano Pietronero. A new metrics for countries' fitness and products' complexity. *Scientific Reports*, 2, 2012.

[40] Eunyoung Choi and Sara Sardari Sayyar. Urban diversity and pedestrian behavior - refining the concept of land-use mix for walkability. *Space Syntax Siymposium Proceedings*, 8, 2011.

[41] John I. Gilderbloom, William W. Riggs, and Wesley L. Meares. Does walkability matter? an examination of walkability's impact on housing values, foreclosures and crime. *Cities*, 42, 2015.

[42] Bela Balassa. Trade liberalisation and "revealed" comparative advantage. *The Manchester School*, 33, 1965.

[43] Sándor Juhász, Gergő Pintér, Ádám Kovács, Endre Borza, Gergely Mónus, László Lőrincz, and Balázs Lengyel. Amenity complexity and urban locations of socio-economic mixing, 2022.

[44] César A. Hidalgo and Ricardo Hausmann. The building blocks of economic complexity. *Proceedings of the National Academy of Sciences of the United States of America*, 106, 2009.

[45] P. K. Sen, Richard H. Lindeman, Peter F. Merenda, and Ruth Z. Gold. Introduction to bivariate and multivariate analysis. *Journal of the American Statistical Association*, 76, 1981.

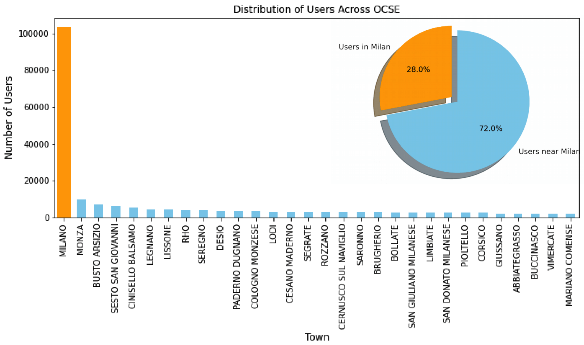

Figure S1 Distribution of users across Milan city and its neighboring regions within the FUA area (Milan area). The histogram showcases the most 20 populated towns within the FUA area. 28% of users are located in Milan city, while the remaining 72% are dispersed across nearby areas

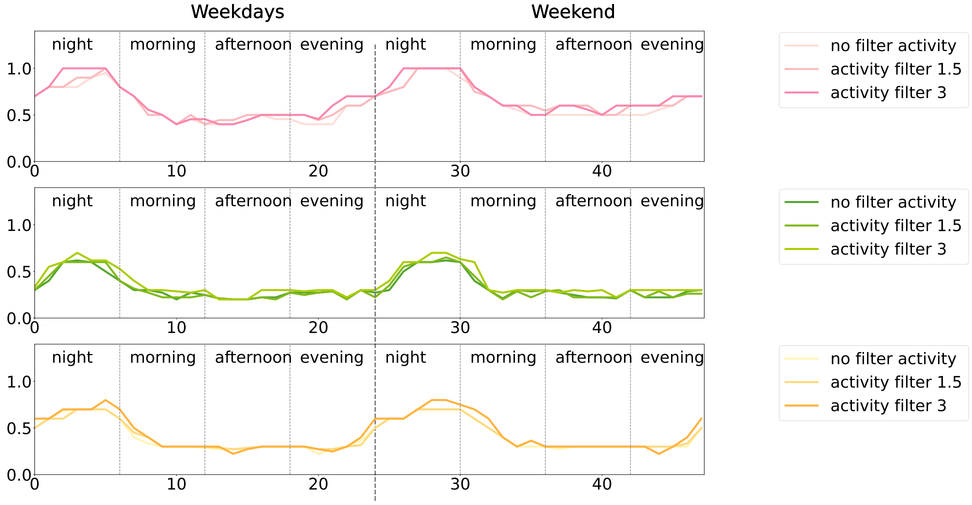

Figure S2 Segregation profile for each ALA cluster over a 48-hour timeframe, encompassing both a typical weekday and a weekend. The data has been processed through several filters to reflect diverse levels of user activity per day, depicted via a spectrum of color gradients. The Gini index is employed to calculate the variations in the segregation measure.

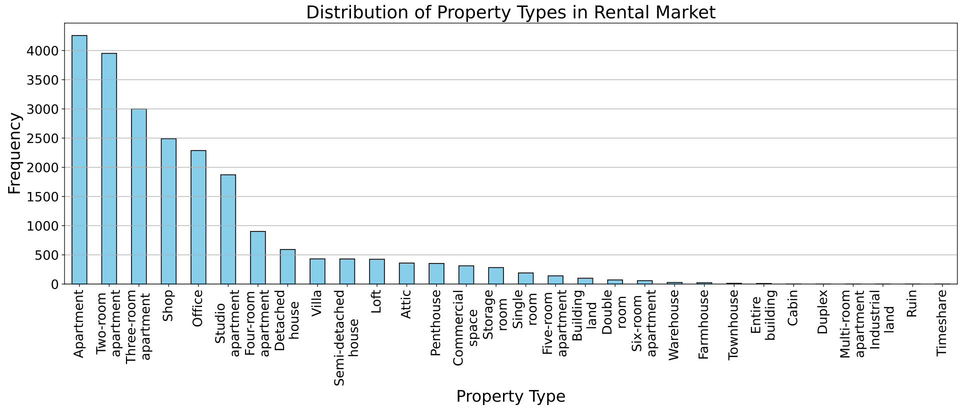

Figure S3 Distribution of different categories of rental properties available in Milan. The chart illustrates the variety of property types.

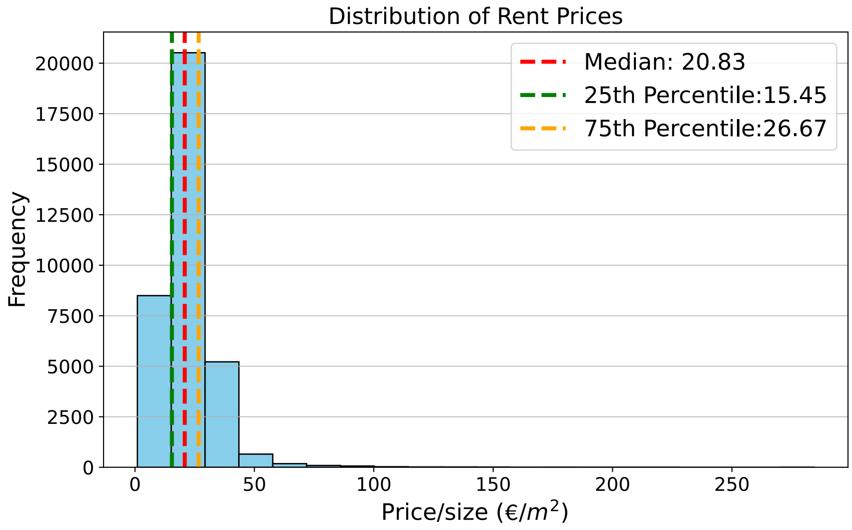

Figure S4 Distribution of rental prices per square meter for habitable residences in Milan. This Figure presents a comprehensive view of the price range and variability, highlighting the economic diversity within the city's real estate market.

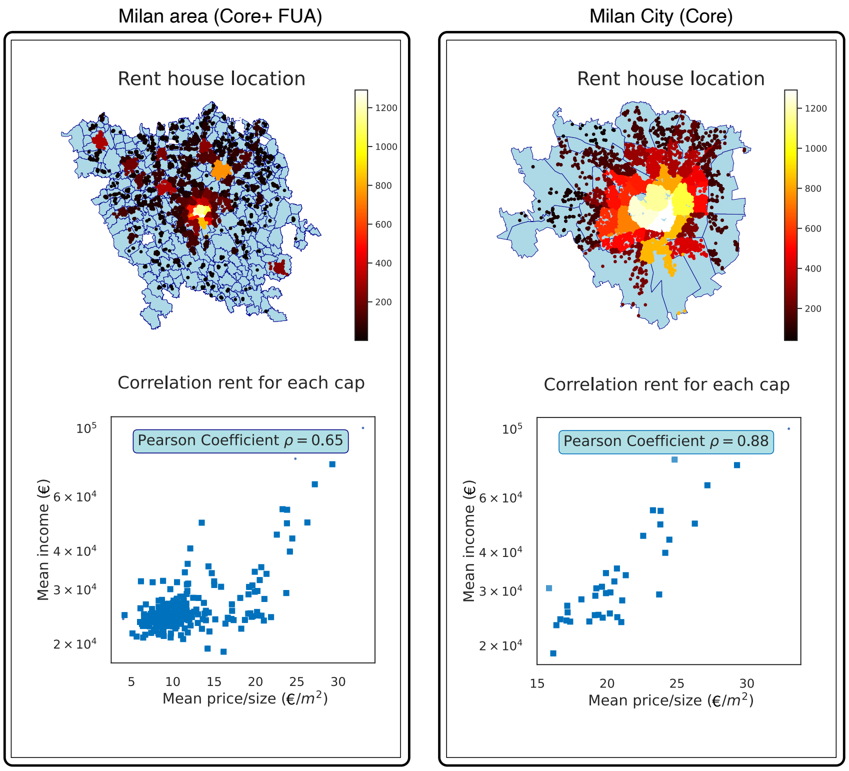

Figure S5 Rent data as a proxy of income. Maps of rented houses in Milan metropolitan area (upper-left) and Milan city (upper-right). Correlation in each zip code of Milan metropolitan area (lower-left) and Milan city (lower-right). Each point in the scatter plot represents a zip code. The x-axis represents the real mean income from census, and the y-axis represents the mean of the price on square meters.

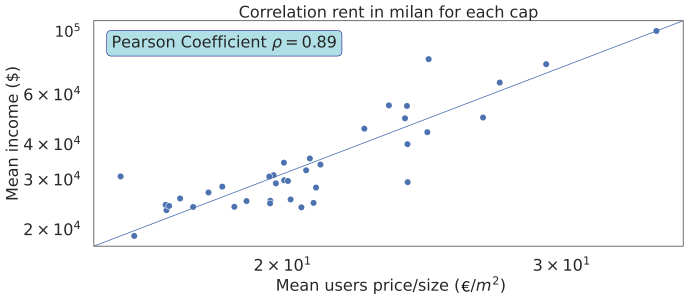

Figure S6 Representativeness of Income. Correlation in each ZIP code of Milan city. Each point in the scatter plot represents a ZIP code. The x-axis denotes the real median income from census data, while the y-axis represents the median value assigned to users (based on the mean rent prices of the ten closest properties to each user's home location).

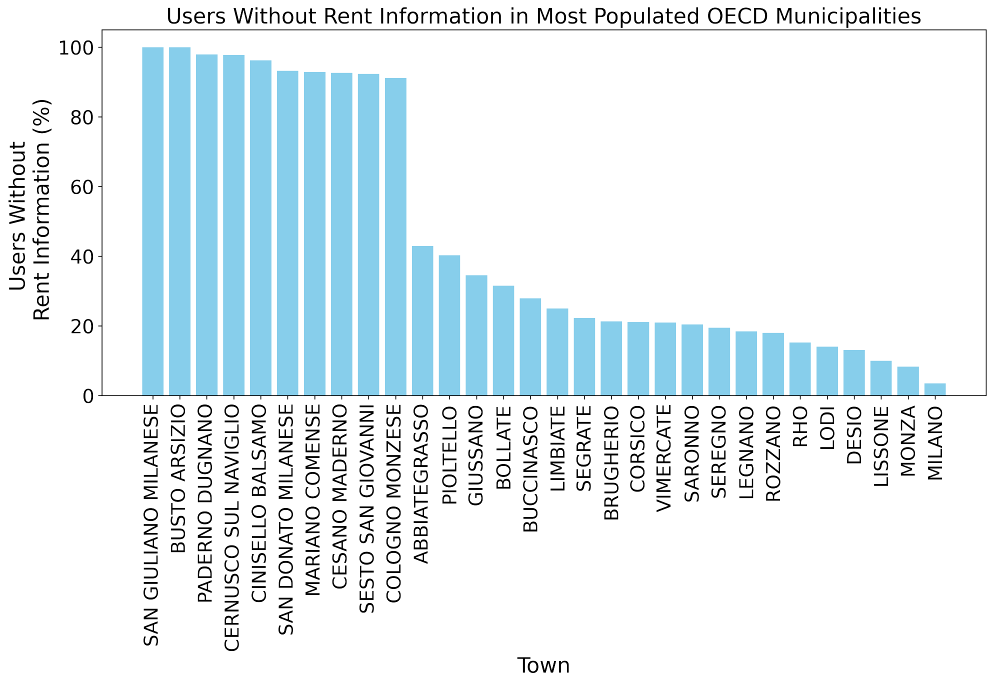

Figure S7 Assigned rental values across the 30 most populous municipalities in Milan area. The majority of municipalities have no users with valid income assignments, highlighting data sparsity outside Milan city (core).

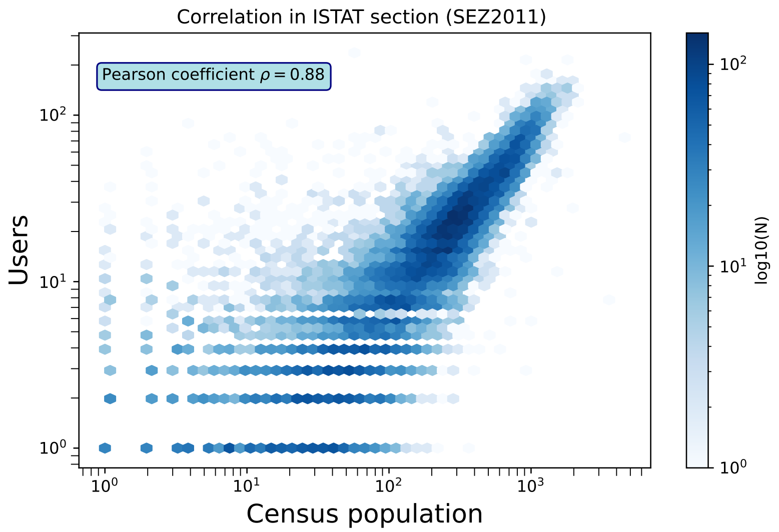

Figure S8 Correlation between the users and the census population in each ISTAT section.

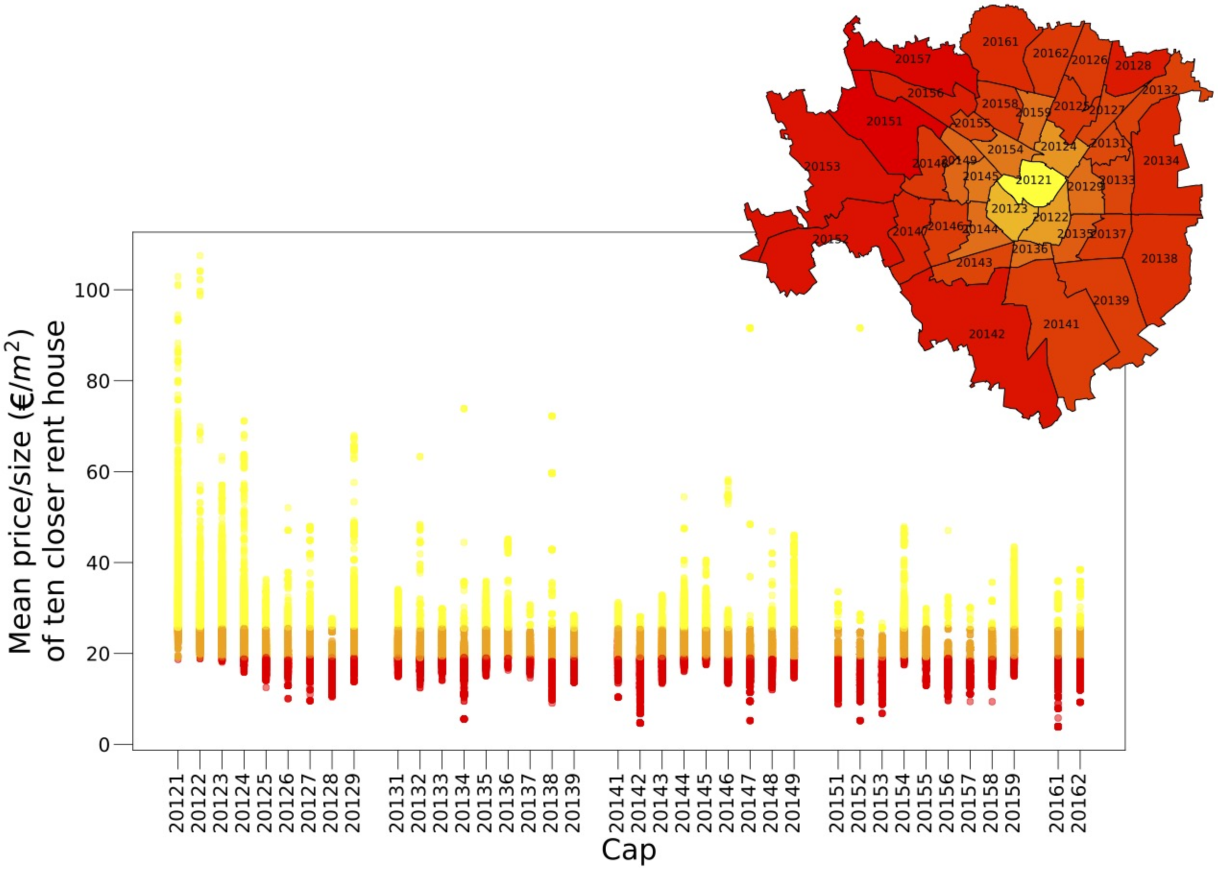

Figure S9 Income categorisation using K-means clustering based on the average price per square meter of the nearest 10 houses within a 200-meter radius from each user's residence, plotted against ZIP codes. The plot exhibits a segregation into high (yellow), medium (orange), and low (red) income groups. Additionally, a spatial map computed using GeoPandas demonstrates the geographical distribution of ZIP codes predominantly occupied by a particular income group, thereby illustrating the spatial arrangement of economic strata within the urban fabric.

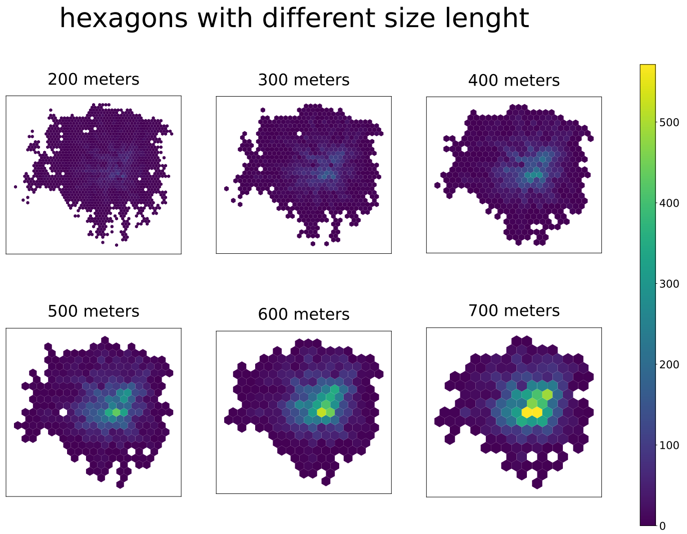

Figure S10 Distribution Maps of Points of Interest (POIs) for Grids of Different Sizes. Each subplot represents a hexagonal grid of a particular side length overlaid on a map, with cells coloured based on the count of POIs contained within. This visual representation illustrates the variance in POI density and distribution across different grid scales.

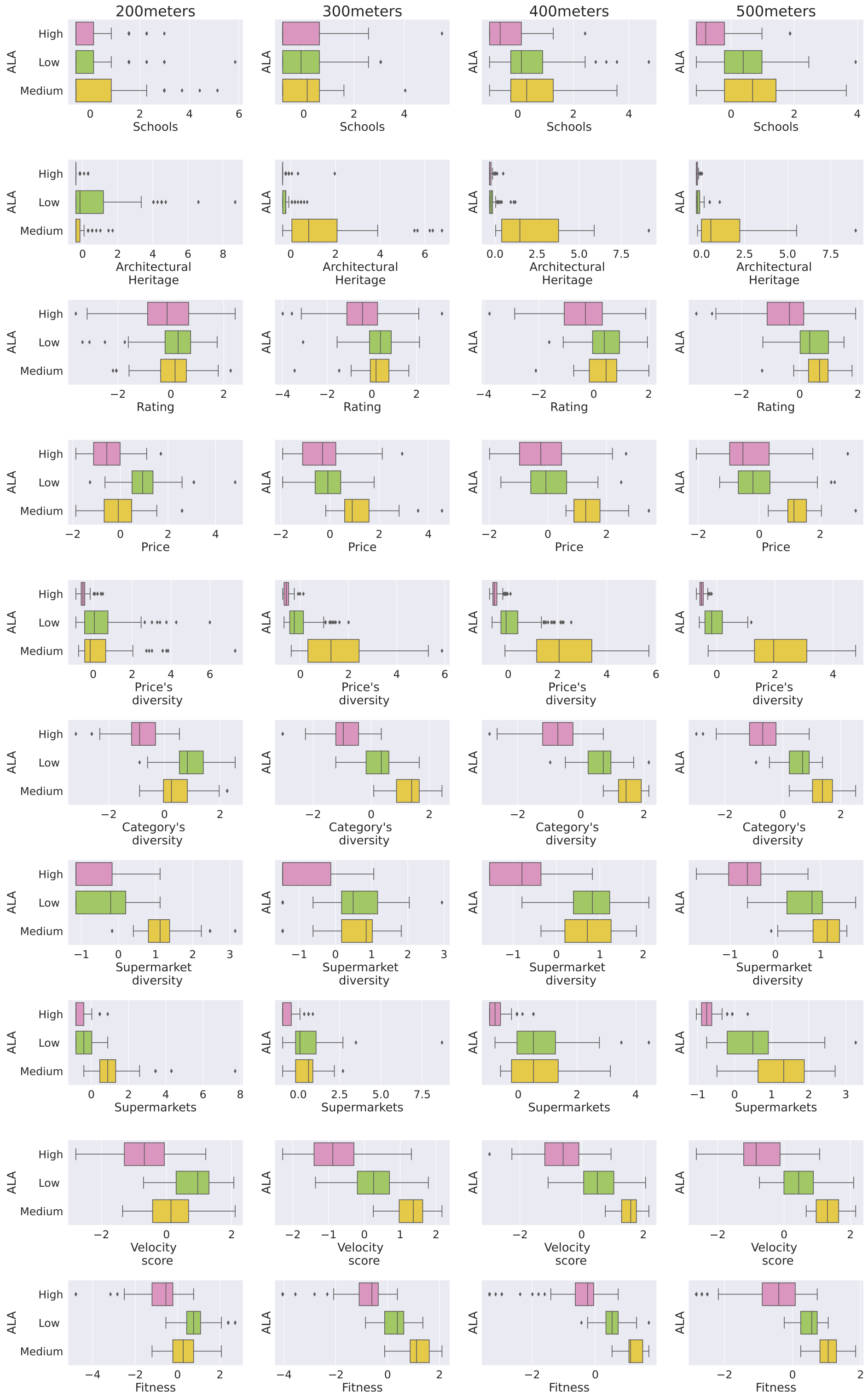

Figure S11 Boxplot illustrating the Z-score distribution of ALA metrics across different hexagonal grid sizes. The metrics encompass architectural heritage, ratings, price, price diversity, schools, category diversity, supermarket diversity, supermarket count, velocity score, and fitness. Each ALA cluster is represented by a distinct colour, and each column corresponds to a unique grid size, showcasing the variability in metric values across neighbourhoods and spatial resolutions.

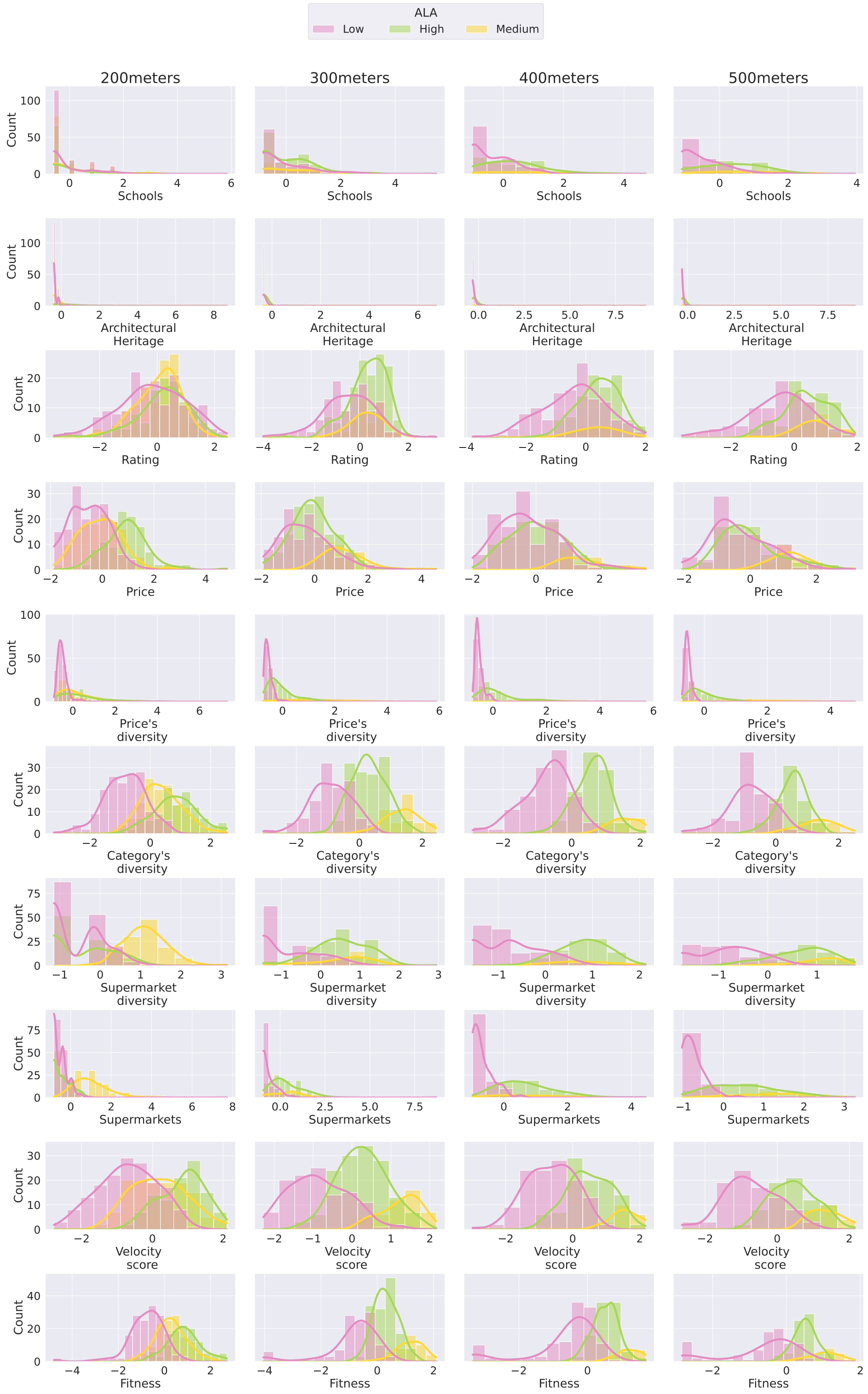

Figure S12 Z-score distribution of ALA metrics across different hexagonal grid sizes. The metrics encompass architectural heritage, ratings, price, price diversity, schools, category diversity, supermarket diversity, supermarket count, velocity score, and fitness. Each ALA cluster is represented by a distinct colour, and each column corresponds to a unique grid size, showcasing the variability in metric values across neighbourhoods and spatial resolutions.

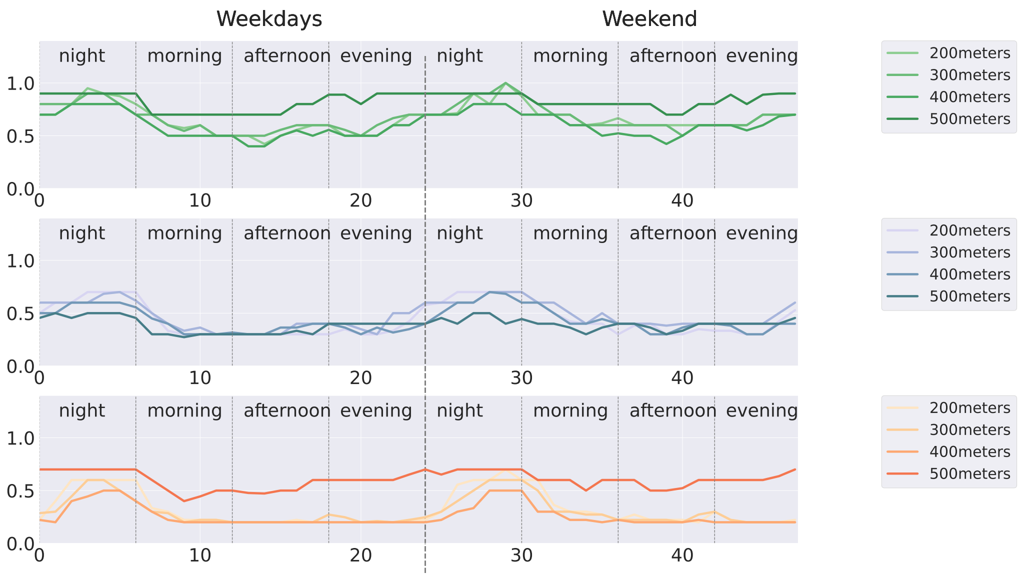

Figure S13 Segregation profile depicted for each hexagonal grid across a 48-hour period (24 hours on a weekday and 24 hours on a weekend). The analysis is segmented into three social mixing clusters: segregated (red), mixed (blue), and inclusive (green). The grid size is expressed through the colour gradients. The segregation measure is computed using the Gini index.

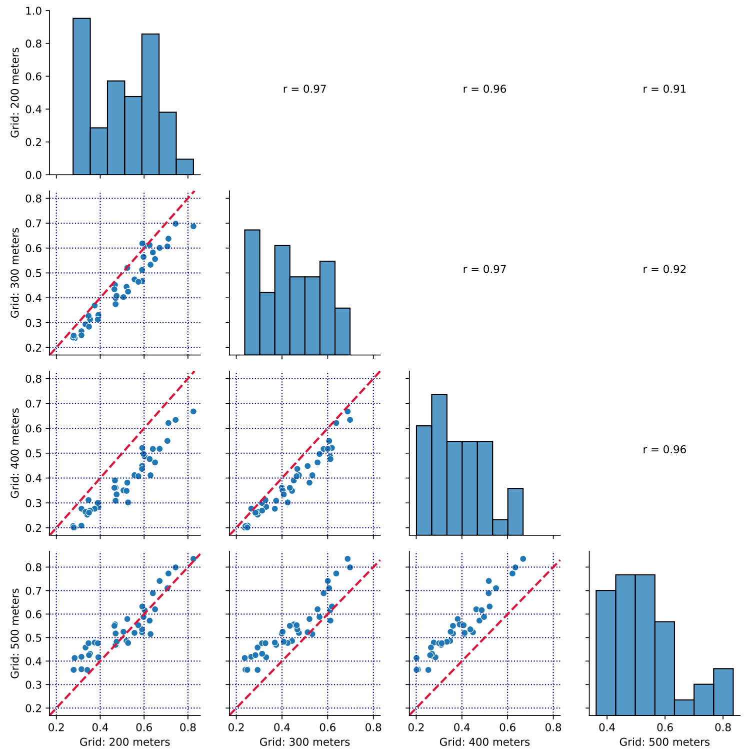

Figure S14 Correlation and Distribution of Segregation Across Hexagonal Grids: correlation matrix illustrating the relation between different grid configurations. The matrix shows correlation coefficients, scatter plots, and distribution along the diagonal. Each point within the scatter plots represents the median segregation value of the grids contained within a specific zip code.

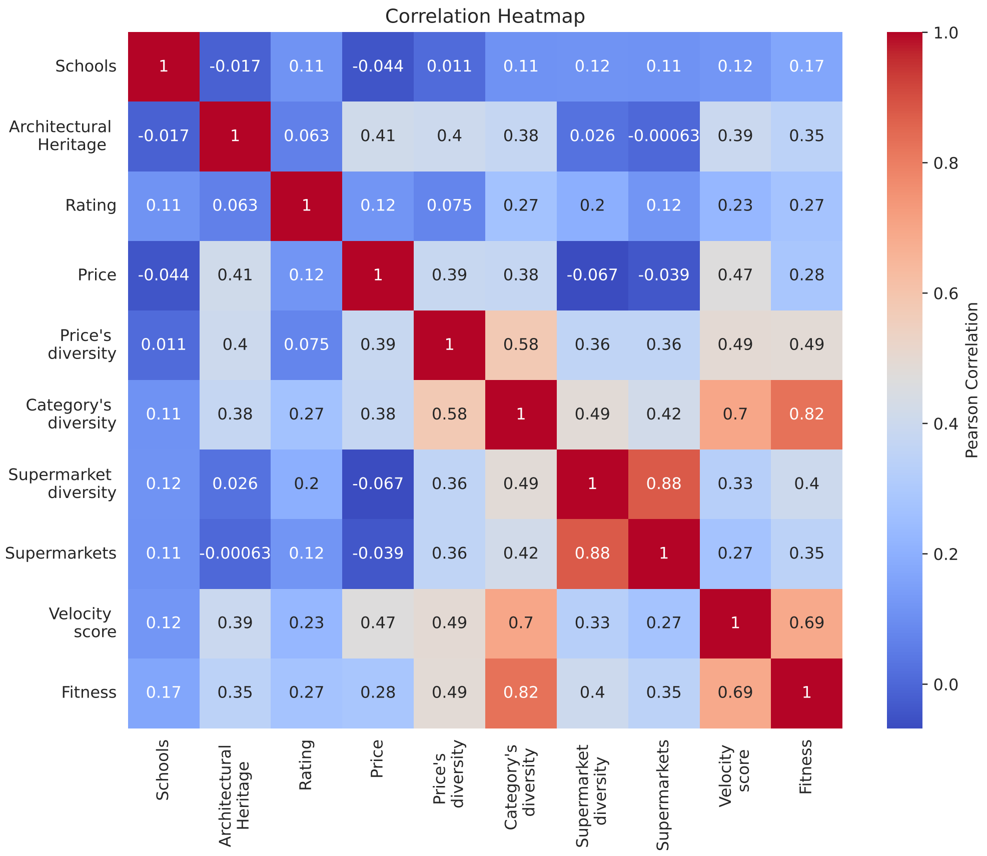

Figure S15 Correlation Between the ALA Metrics.

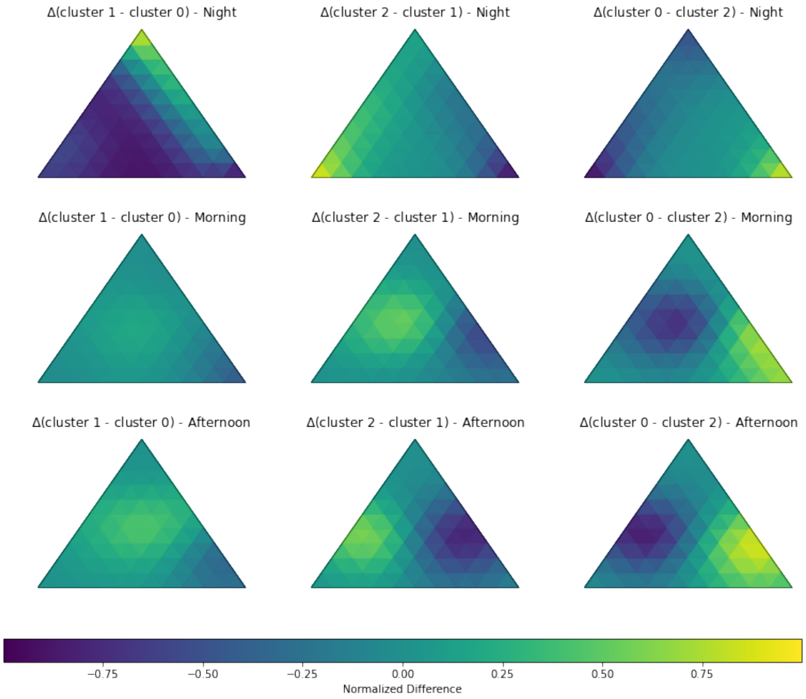

Figure S16 Ternary plot showing the differences between each income group. Lighter colours indicate positive values, while darker shades represent negative values. This visualisation helps in understanding the difference in income distribution across different clusters.

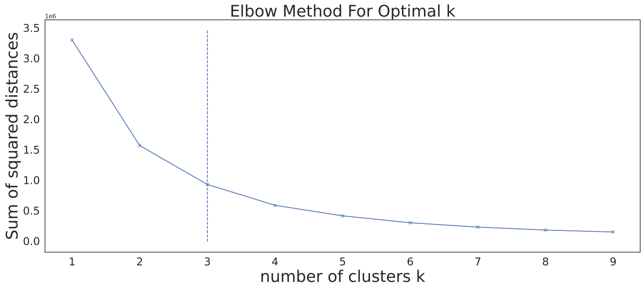

Figure S17 Elbow method analysis for income group clusterisation. The elbow point suggests the optimal number of clusters for categorizing income groups.

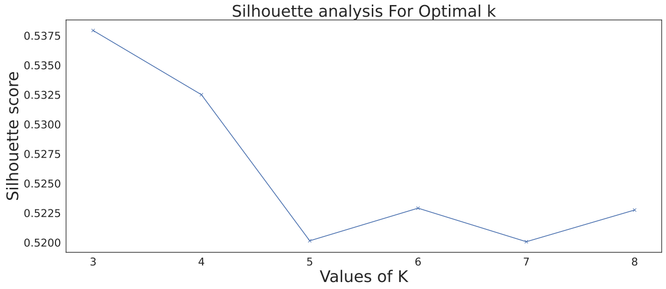

Figure S18 Silhouette score analysis for income group clusterisation. This analysis aids in validating the coherence and separation of the chosen clusters.

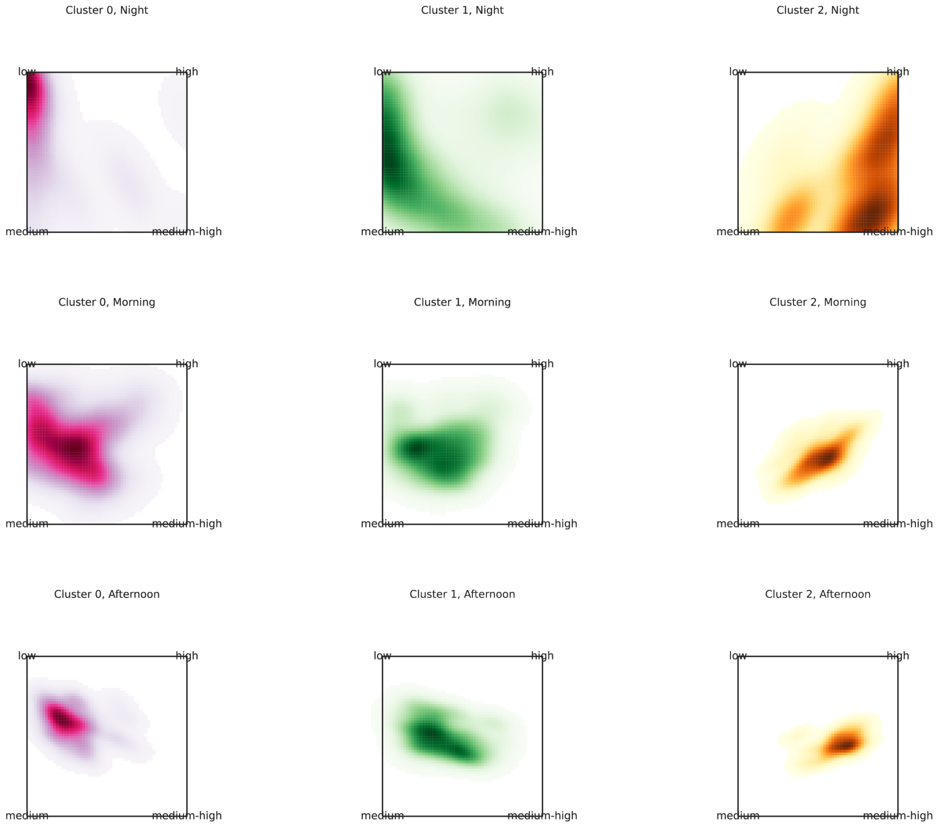

Figure S19 Square-form ternary plot adapted from the main paper to represent 4 income groups. This visualisation format is used to accommodate an additional income group, resulting in a square layout. Each corner of the square represents one of the four income groups, with columns indicating temporal clusters and rows representing temporal windows.

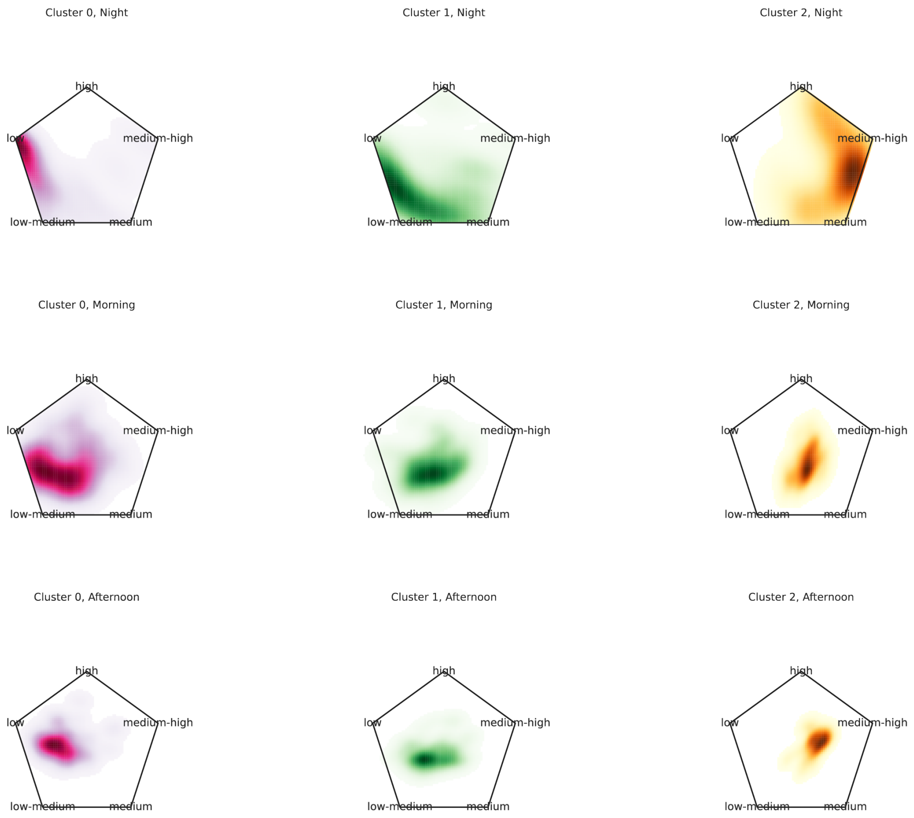

Figure S20 Pentagon-form ternary plot adapted from the main paper to represent 5 income groups. This visualisation format is used to accommodate an additional income group, resulting in a pentagon layout. Each vertex of the pentagon represents one of the five income groups, with columns indicating temporal clusters and rows depicting temporal windows.

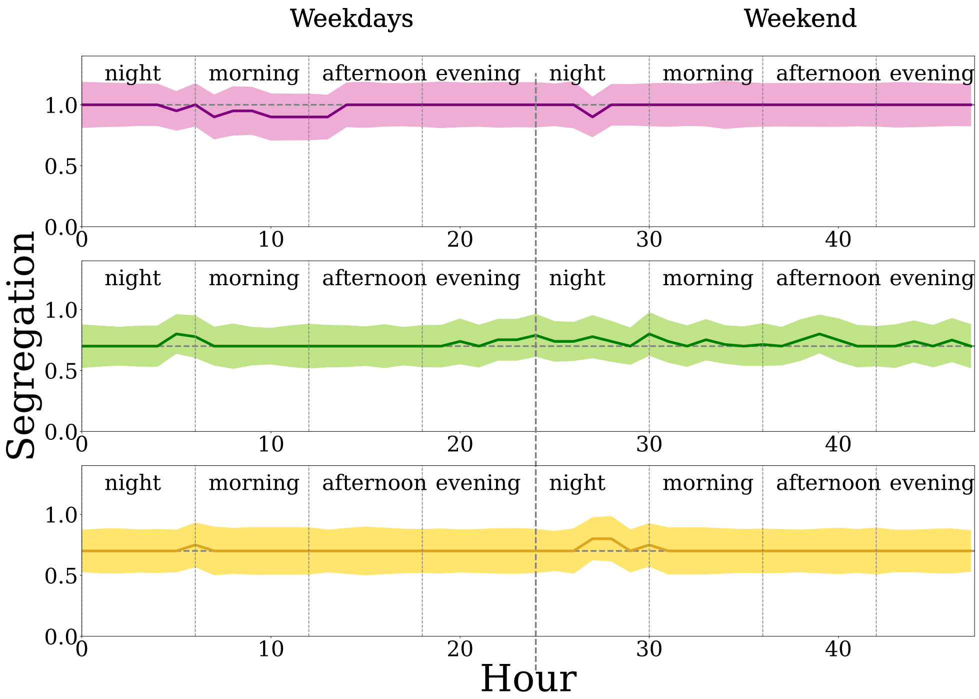

Figure S21 Segregation profile depicted within the ALA clusters based solely on stops made at users' residences. The analysis spans a 48-hour period (24 hours on a weekday and 24 hours on a weekend), with segregation being measured using the Gini index. The segregation is segmented into three clusters: high (yellow), medium (green), and low (pink).

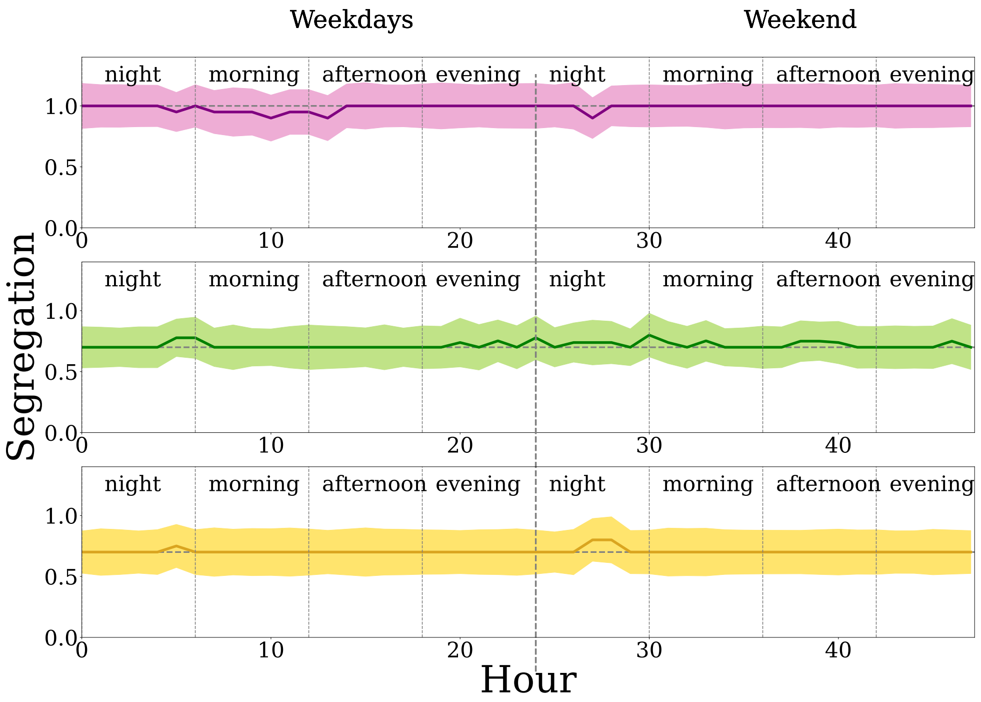

Figure S22 Segregation profile depicted within the ALA clusters based solely on stops made at users' workplaces. The analysis spans a 48-hour period (24 hours on a weekday and 24 hours on a weekend), with segregation being measured using the Gini index. The segregation is segmented into three clusters: high (yellow), medium (green), and low (pink).

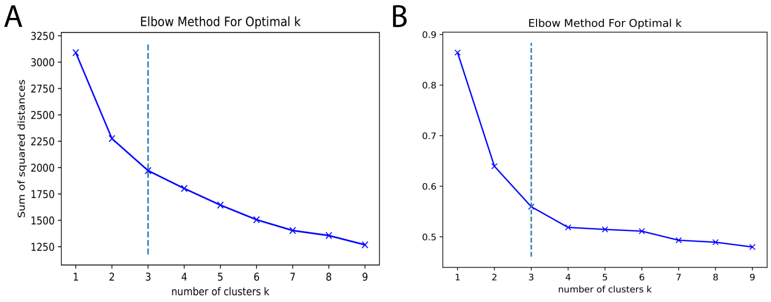

Figure S23 Elbow method analysis for hexagon clusterisation. The elbow point suggests the optimal number of clusters for categorizing hexagons in ALA metrics (**A**) and in temporal mixing profile (**B**).

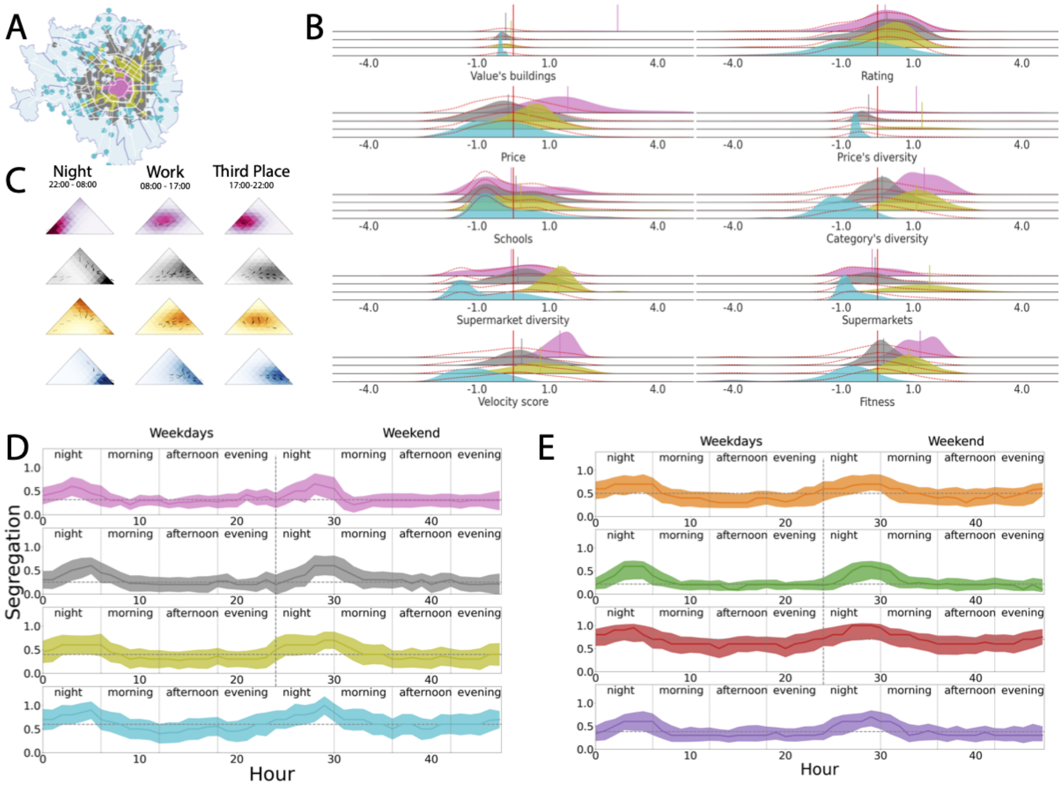

Figure S24 Analysis of the ALA clusters for 4 clusters. **A**: Map of ALA clusters. **B**: Distribution of ALA metrics per cluster. **C**: Ternary plot for the 4 ALA clusters. **D**: Segregation trends within the ALA clusters over weekdays and weekends. **E**: Segregation trends for the temporal mixing clusters.

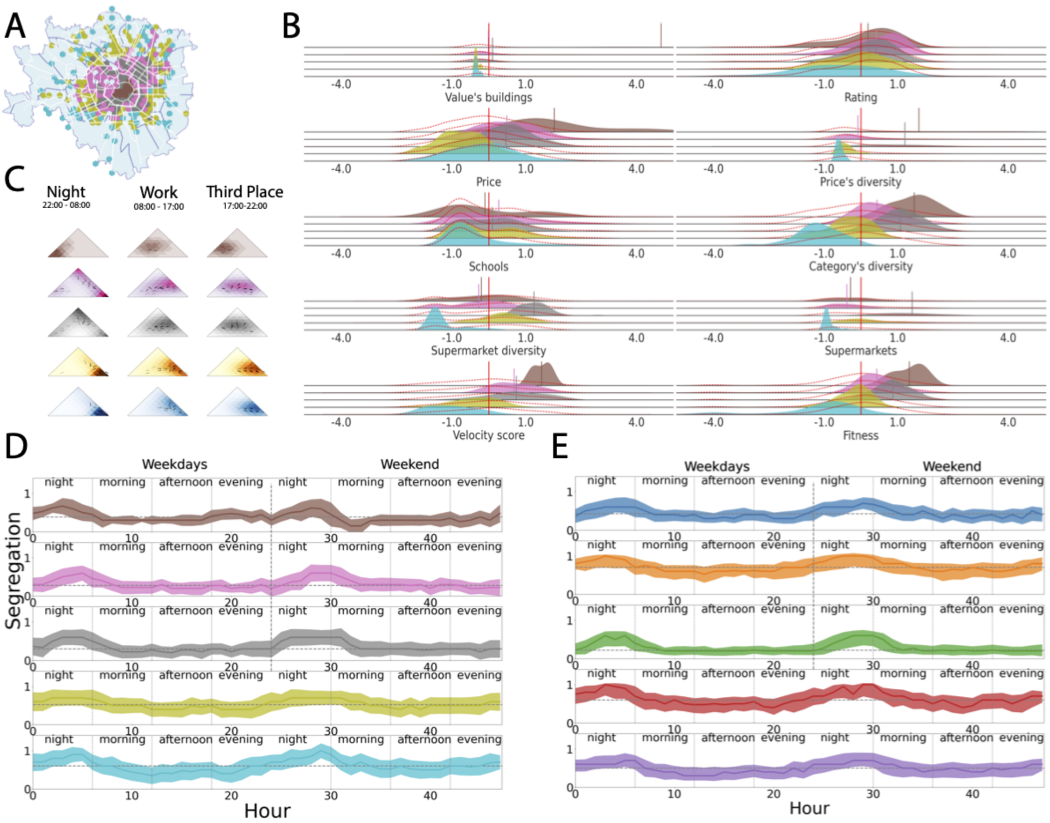

Figure S25 Analysis of the ALA clusters for 5 clusters. **A**: Map of ALA clusters. **B**: Distribution of ALA metrics per cluster. **C**: Ternary plot for the 5 ALA clusters. **D**: Segregation trends within the ALA clusters over weekdays and weekends. **E**: Segregation trends for the temporal mixing clusters.

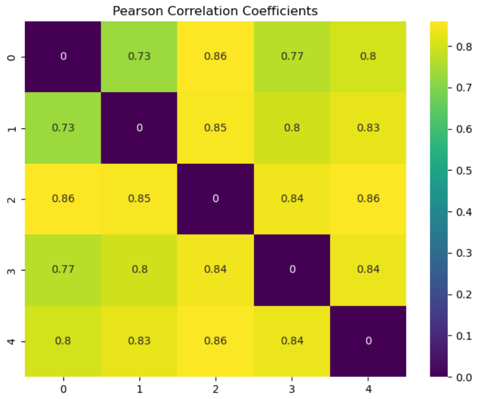

Figure S26 Heatmap of Pearson Correlation Coefficients between Different Clusters. This heatmap visualises the pairwise Pearson correlation coefficients for curves derived from each cluster in the dataset. Each cell represents the correlation coefficient between two clusters, with values closer to 1 indicating a higher degree of similarity in the patterns of the respective curves.

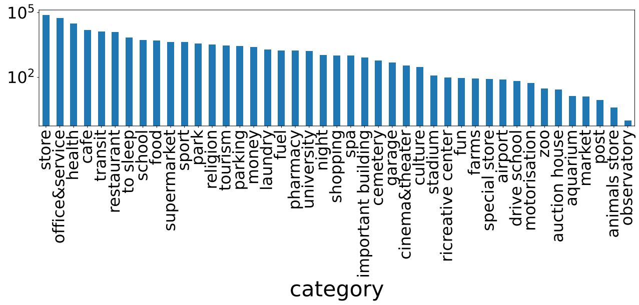

Figure S27 Number of categories in Google Place dataset

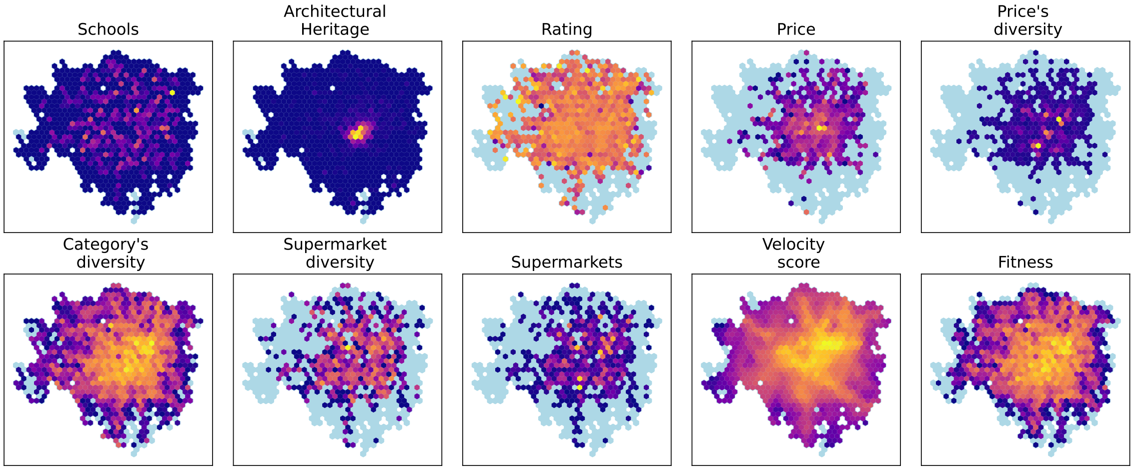

Figure S28 Map distributions for each hexagon representing the ten ALA metrics across Milan. Each map, computed using Geopandas, illustrates the spatial distribution of a particular feature within the city. The colour corresponds to the value of the feature in the hexagon, with the yellow indicating higher values and the blue having lower values.

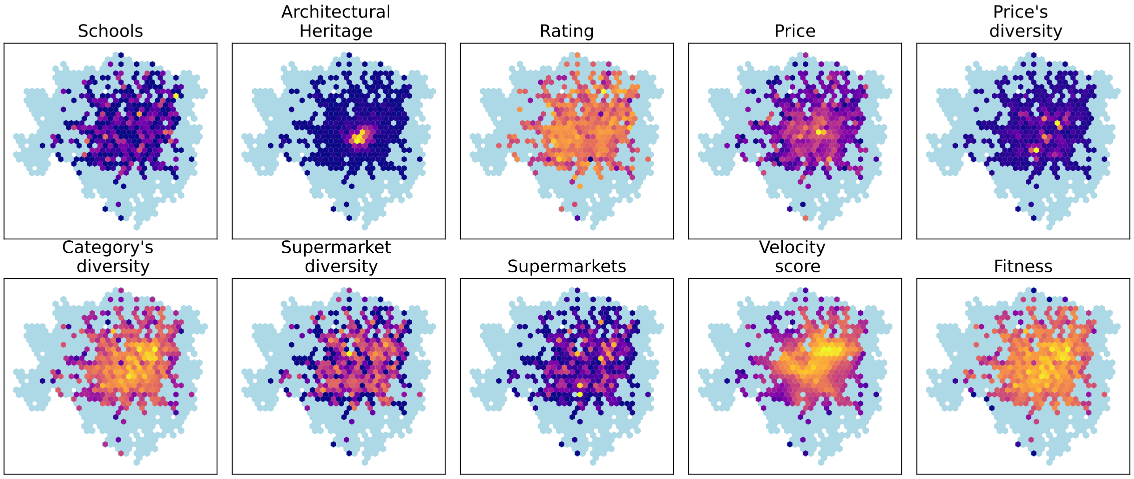

Figure S29 Map distributions for each hexagon representing the ten ALA metrics across Milan, considering only the hexagons where all ALA features are non-null. Each map, computed using Geopandas, illustrates the spatial distribution of a particular feature within the city. The colour corresponds to the value of the feature in the hexagon, with the yellow indicating higher values and the blue having lower values.

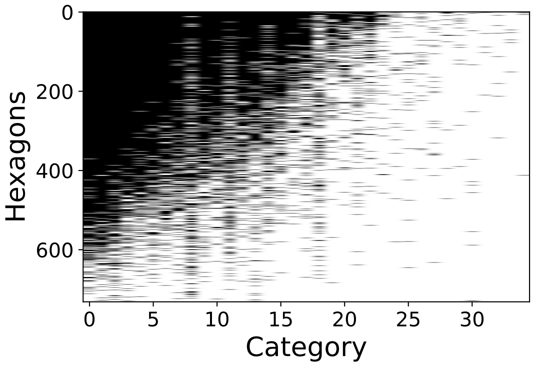

Figure S30 The (bi-)adjacency matrix of the bipartite hexagon-categories network where the rows (hexagons) and columns (categories) have been sorted according to the fitness-complexity algorithm

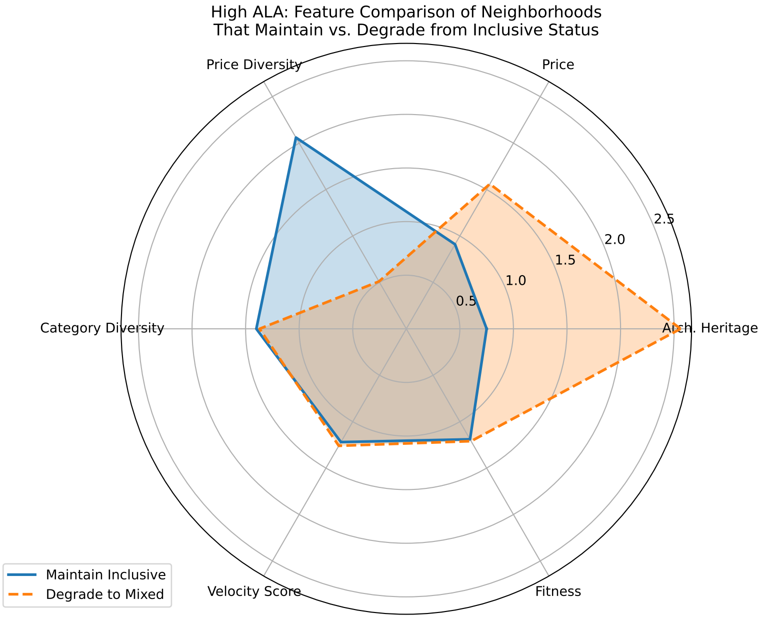

Figure S31 Feature comparison of high-ALA neighborhoods that maintain inclusive status versus those that degrade to mixed status. The radar plot compares key urban characteristics between two types of high-quality neighborhoods: those that remain socially inclusive throughout all time periods (solid blue line) and those that become more segregated during non-working hours (dashed orange line).
